# Supplementary material for: The PERK Inhibitor GSK2606414 Enhances Reovirus Infection in Head and Neck Squamous Cell Carcinoma via an ATF4-Dependent Mechanism
Source: Mol Ther Oncolytics. 2020 Jan 17;16:238–49. doi: 10.1016/j.omto.2020.01.001 (PMC7047134; doi:10.1016/j.omto.2020.01.001)
Supplement: Document S1. Figures S1–S3 and Tables S1 and S2 [file mmc1.pdf]

OMTO, Volume *16*

Supplemental Information

The PERK Inhibitor GSK2606414 Enhances Reovirus Infection in Head and Neck Squamous Cell Carcinoma via an ATF4-Dependent Mechanism

Martin McLaughlin, Malin Pedersen, Victoria Roulstone, Katharina F. Bergerhoff, Henry G. Smith, Harriet Whittock, Joan N. Kyula, Magnus T. Dillon, Hardev S. Pandha, Richard Vile, Alan A. Melcher, and Kevin J. Harrington

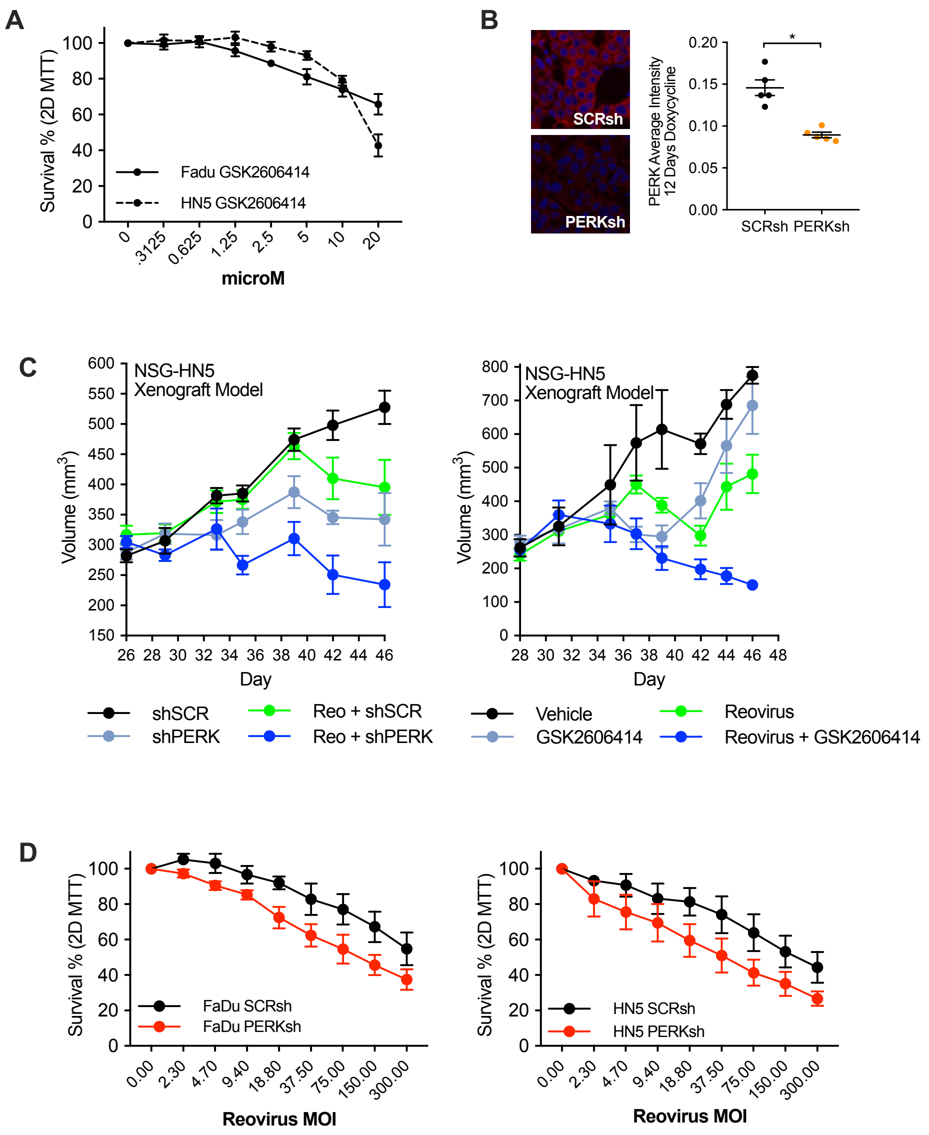

Supplementary Figure S1. Additional viability and growth curves to data in Figure 1. (A) Survival data for GSK2606414 alone in FaDu and HN5 cell lines measured at 72 h by MTT assay. (B) $2x10^6$ HN5 cells stably expressing lentiviral Tet-pLKO-puro were injected subcutaneously into NSG mice. HN5 cells contained scrambled shRNA (SCRsh) or PERK targeting shRNA (PERKsh). All mice received 50 mg/Kg doxycycline daily by gavage. After 12 days FFPE tumors were sectioned and stained by IHF to validate PERK knockdown. PERK levels were measured by automated image quantification using cell profiler. Example images of staining quality are shown for each. Data ±SEM with with statistical analysis by unpaired t-test *P < 0.05. Individual data points correspond to values for one tumor. (C) Corresponding volume data in mm^3 from figures 1F and 1G. (D) Survival as measured by MTT assay for reovirus in FaDu and HN5 cells with scrambled or PERK shRNA. Cells were treated with 400 g/mL doxycycline for 96 h before the addition of reovirus. Cells were maintained in doxycycline until the end of the assay at 72 h. Data shown in both A and D are from a minimum of three independent experiments.

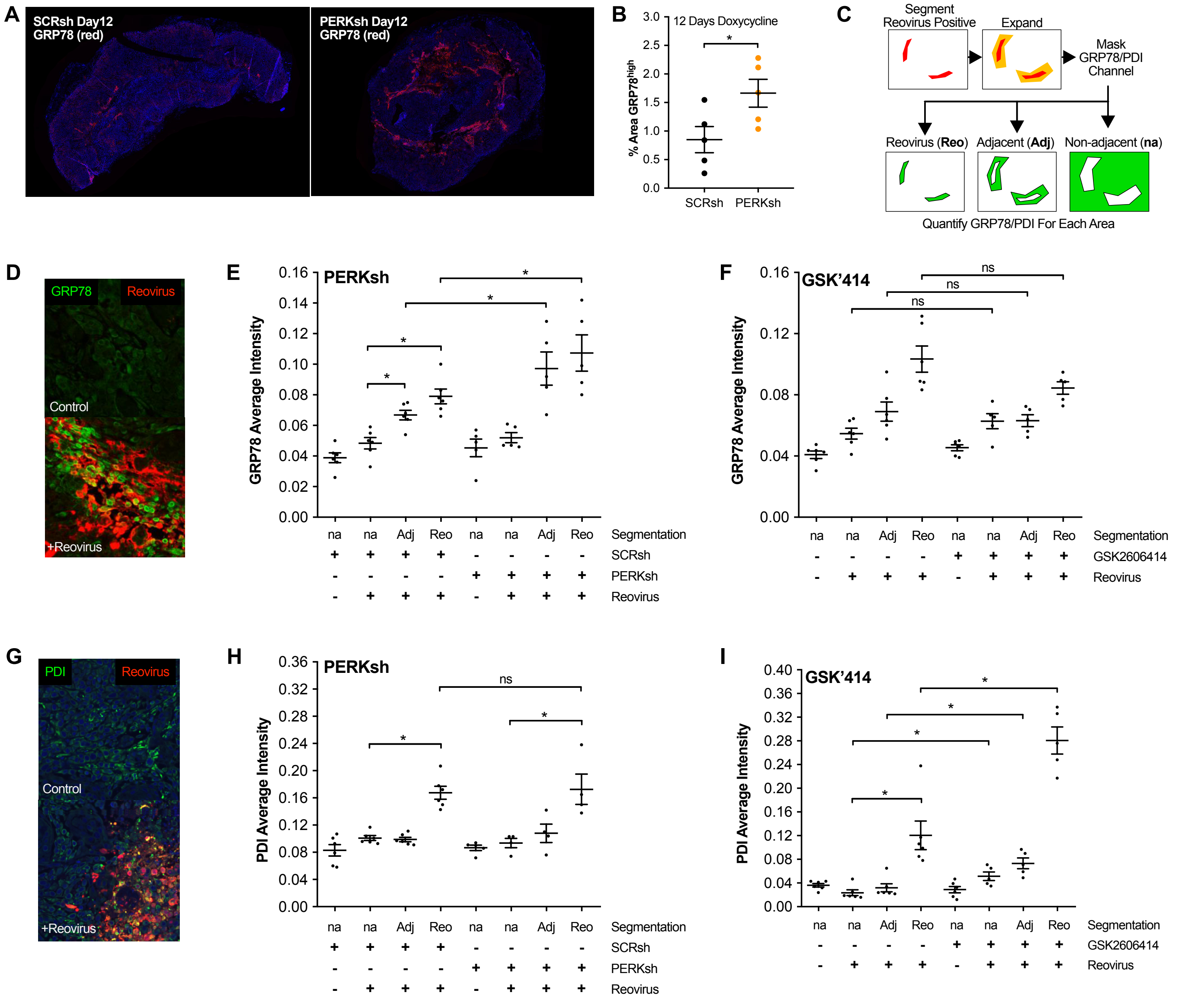

Supplementary Figure S2. Modulation of reovirus induced GRP78 and PDI *in vivo* by PERK knockdown or GSK2606414. (A) $2x10^6$ HN5 cells stably expressing lentiviral Tet-pLKO-puro were injected subcutaneously into NSG mice. HN5 cells contained scrambled shRNA (SCRsh) or PERK targeting shRNA (PERKsh). All mice received doxycycline daily by gavage. After 12 days FFPE tumors were sectioned and stained by IHF to assess changes to levels of the ER chaperone GRP78. (B) GRP78 high levels were quantified as in figure 3. (C) FFPE *in vivo* tumors at the end of experiments in figures 1F and 1G were co-stained for reovirus and GRP78 or PDI by IHF and imaged using the Vectra 3 imaging system from Perkin Elmer. Images were subject to automated image analysis using cell profiler. A schematic overview of the segmentation used in panels D-I to quantify GRP78 and PDI in areas positive for reovirus (Reo), adjacent to reovirus (Adj) and outside these two areas classified as non-adjacent (na). The area expanded by 10 microm around reovirus positivie areas equals approximately 1-2 cell widths immediately adjacent to reovirus infected areas with each Vectra image 525x700 microm in total. (D) Representative images of reovirus mu1C and GRP78 staining. (E,F) Comparison between the impact of PERK knockdown or GSK2606414 on GRP78 levels induced by reovirus infection. (G) Representative images of reovirus mu1C and PDI staining. (H,I) Comparison between the impact of PERK knockdown or GSK2606414 on PDI levels induced by reovirus infection. All panel data ±SEM with statistical analysis by unpaired t-test *P < 0.05. Individual data points correspond to values for one tumor.

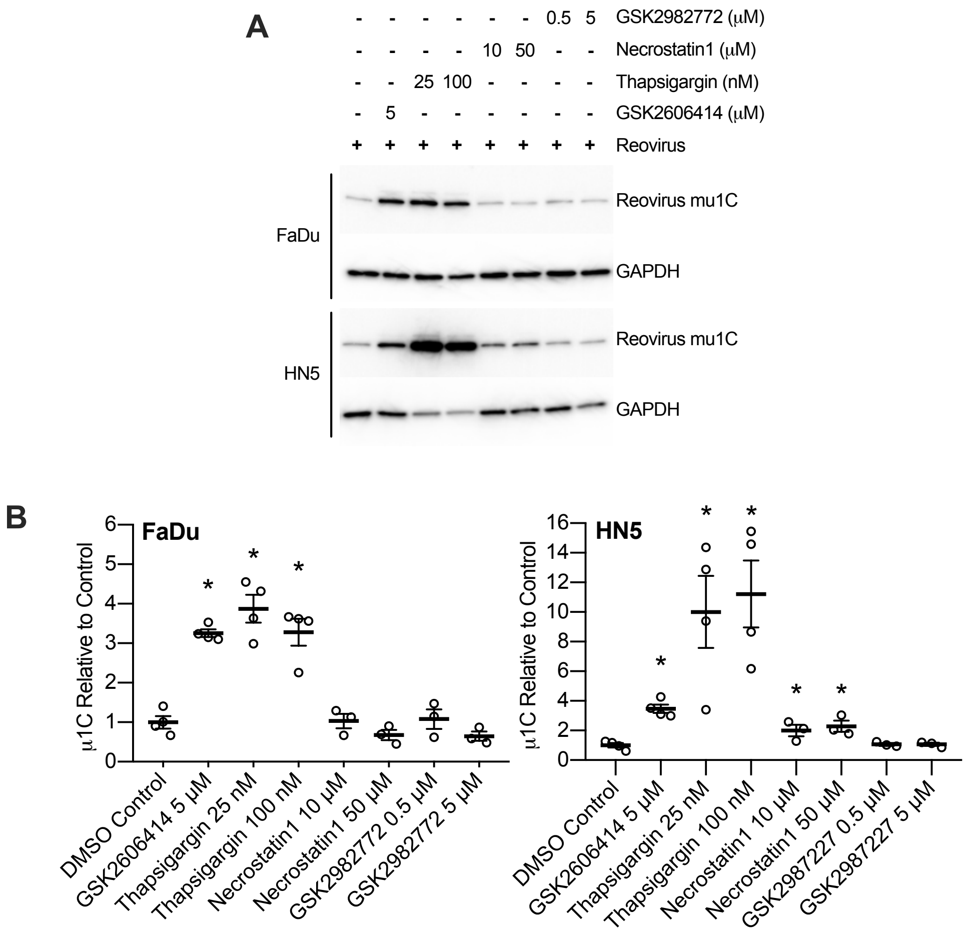

Supplementary Figure S3. Comparison of GSK2606414, Thapsigargin and RIPK1 inhibitors Necrostatin1 and GSK2982772 on reovirus capsid levels. (A) FaDu and HN5 cells were treated with reovirus in combination with DMSO only control, GSK2606414, or the RIPK1 inhibitors necrostatin1 and GSK2982772. Cell lysates were collected after 48 h and probed by western blot for the reovirus capsid protein mu1C. (B) Densitometry quantification of reovirus mu1C from western blots. Data is corrected for GAPDH levels and normalised to the average of controls. Data ±SEM of a minimum of three independent experiments with statistical analysis by unpaired t-test *$P < 0.05$.

Supplementary Table 1. Primers used for UPR live cell reporter cloning.

Reporter	Primer Name	Sequence
IRE1 endo	XBP1intron GFPFLAGforward	GTTGGATCCGCCACCATGCCAGTGGCCGGGTCTGCTGAGTCCGCAGCACTCAGAC TACGTGCACCTCTGCAGCAGGTGCAGGCCCAGTTGTCGTGAGCAAGGGCGAGGAG
IRE1 endo	XBP1intron GFPFLAGreverse	AATCAGGCGGCCGCTTACTTGTCGTCATCGTCTTTGTAGTCC
ATF4 prom	ATF4 forward	TTTCCAGAGTTTAGGAGGAGCCATG
ATF4 prom	ATF4 forward + 5' Overlap pHR	CAGAGATCCAGTTTGGATCGATAAGCTTGATATCGAATTCTTTCCAGAGTTTAGGAG GAGCCATG
ATF4 prom	ATF4 reverse	CATGTTGCGGTGCTTTGCTGGAA
ATF4 prom	ATF4 reverse + 3'Overlap mCherry	TGATGATGGCCATGTTATCCTCCTCGCCCTTGCTCACCATGTTGCGGTGCTTTGCTG GAA
ATF4 prom	mCherryMYC forward	ATGGTGAGCAAGGGCGAG
ATF4 prom	mCherryMYC reverse + 3' Overlap pHR	AGCTTGCATGCCTGCAGGTCGACTCTAGAGTCGCGGCCGCTTACAGATCCTCTTCT GAGATGAGTTTTTGTTCC
ATF6 bind	p5xATF6 forward	TAGGTCGAATTCCTCGAGACAGGTGCTGACG
ATF6 bind	p5xATF6 reverse	AATCAGGGATCCGGAGATCCTCTAGAGTCGACTCTAG

Supplementary Table 2. Primers for ATF4 transcript variants 1 and 2. Exon-Exon boundary indicated by /.

Variant	Primer Name	Sequence
2	ATF4 var2 forward	AGCCATGGC/GCTTCTCAC
	ATF4 var2 reverse	TGGAACACACAGCTACAGCA
1&2	ATF4 var1 and var2 forward	ATGGCGTATTAGGGGCAG
	ATF4 var1 and var2 reverse	AAGGCATCCT/CCTTGCTGTT
