# Supplementary material for: The PERK Inhibitor GSK2606414 Enhances Reovirus Infection in Head and Neck Squamous Cell Carcinoma via an ATF4-Dependent Mechanism
Source: Mol Ther Oncolytics. 2020 Jan 17;16:238–49. doi: 10.1016/j.omto.2020.01.001 (PMC7047134; doi:10.1016/j.omto.2020.01.001)
Supplement: Document S2. Article plus Supplemental Information [file mmc2.pdf]

Molecular Therapy
Oncolytics
Original Article

AMERICAN SOCIETY of GENE & CELL THERAPY

The PERK Inhibitor GSK2606414 Enhances Reovirus Infection in Head and Neck Squamous Cell Carcinoma via an ATF4-Dependent Mechanism

Martin McLaughlin,[1] Malin Pedersen,[1] Victoria Roulstone,[1] Katharina F. Bergerhoff,[1] Henry G. Smith,[1] Harriet Whittock,[1] Joan N. Kyula,[1] Magnus T. Dillon,[1] Hardev S. Pandha,[2] Richard Vile,[3] Alan A. Melcher,[1] and Kevin J. Harrington[1]

[1]The Institute of Cancer Research, London, UK; [2]University of Surrey, Guildford, UK; [3]Mayo Clinic, Rochester, MN, USA

Reovirus type 3 Dearing (reovirus) is a tumor-selective oncolytic virus currently under evaluation in clinical trials. Here, we report that the therapeutic efficacy of reovirus in head and neck squamous cell cancer can be enhanced by targeting the unfolded protein response (UPR) kinase, protein kinase R (PKR)-like endoplasmic reticulum kinase (PERK). PERK inhibition by GSK2606414 increased reovirus efficacy in both 2D and 3D models *in vitro*, while perturbing the normal host cell response to reovirus-induced endoplasmic reticulum (ER) stress. UPR reporter constructs were used for live-cell 3D spheroid imaging. Profiling of eIF2a-ATF4, IRE1a-XBP1, and ATF6 pathway activity revealed a context-dependent increase in eIF2a-ATF4 signaling due to GSK2606414. GSK2606414 blocked eIF2a-ATF4 signaling because of the canonical ER stress agent thapsigargin. In the context of reovirus infection, GSK2606414 induced eIF2a-ATF4 signaling. Knockdown of eIF2a kinases PERK, GCN2, and PKR revealed eIF2a-ATF4 reporter activity was dependent on either PERK or GCN2. Knockdown of ATF4 abrogated the GSK2606414-induced increase in reovirus protein levels, confirming eIF2a-ATF signaling as key to the observed phenotype. Our work identifies a novel approach to enhance the efficacy and replication of reovirus in a therapeutic setting.

INTRODUCTION

Reovirus type 3 Dearing (abbreviated hereafter as reovirus) is an immuno-oncolytic virus under active clinical development. It has been granted orphan drug status by the FDA for malignant glioma. It is a segmented, double-stranded RNA virus with 10 genome segments. Viral proteins provide self-sufficiency in relation to cellular entry via JAM1 and β1-integrins, endosomal escape allowing entry to the cytoplasm, and viral genomic replication.[1,2] Reovirus is dependent on the translational machinery of infected cells for protein synthesis following release of viral RNA into the cytoplasm.

Early studies indicated reovirus tumor selectivity was linked to inactivation of the protein kinase R (PKR)-mediated anti-viral response driven by oncogenic transformation events.[3,4] However, the role of PKR inactivation is debated within the field with evidence of reovirus sensitivity being PKR independent.[5,6] PKR forms part of the cellular integrated stress response (ISR). The ISR centers on the phosphorylation of eIF2a on Ser51 by the kinases PKR, PKR-like endoplasmic reticulum kinase (PERK), general control non-derepressible 2 (GCN2), and Heme-regulated initiation factor 2 alpha kinase (HRI).[7] Phosphorylation prevents eIF2a forming the active GTP-bound state and ternary complex formation with Met-tRNA and the 40S ribosome. This phosphorylation can be induced by anti-viral recognition of dsRNA by PKR, amino acid deprivation via GCN2, heme deprivation via HRI, or endoplasmic reticulum (ER) stress signaling via PERK.

PERK is a component of both the ISR and the unfolded protein response (UPR). The UPR is a tripartite cellular response composed of the ER transmembrane proteins PERK, IRE1a, and ATF6.[8,9] PERK phosphorylation of eIF2a decreases global protein translation while increasing translation of specific mRNA transcripts, such as ATF4, due to regulatory upstream open reading frames.[10,11] IRE1a ribonuclease activity splices an unconventional 26-nt intron in XBP1 mRNA leading to translation of active spliced-XBP1.[12] ATF6 activation is driven by trafficking to the Golgi where cleavage releases the soluble cytoplasmic transcription factor ATF6(N).[13] Through these three cytoplasmic transcription factors, UPR induction acts to resolve stress and restore normal protein homeostasis in the ER.

Although PKR has been shown to play a role in some, but not all, reovirus-permissive cells,[5,14] modulation of global protein synthesis may still occur because of other kinases of the ISR, such as GCN2 and PERK. Studies have shown that reovirus induces ER stress, and that this could be targeted as a strategy to increase reovirus efficacy. Early publications in this area showed reovirus efficacy increased in combination with the proteasomal inhibitor bortezomib.[15,16] More recently, BRAF-MEK inhibition sensitized melanoma to reovirus via increased ER stress.[17] Basic research into reovirus biology

Received 25 June 2019; accepted 2 January 2020;
https://doi.org/10.1016/j.omto.2020.01.001.

Correspondence: Martin McLaughlin, The Institute of Cancer Research, London, UK.
E-mail: martin.mclaughlin@icr.ac.uk

This is an open access article under the CC BY license (http://creativecommons.org/licenses/by/4.0/).

indicates that despite a cytoplasmic life cycle, reovirus exerts profound effects on the ER. Reovirus produces viral factories through reorganization of cell membranes.[18] Neo-organelle formation is driven by the non-structural proteins σNS and μNS[19] that act to remodel the ER, forming viral inclusion structures.

Given the ability of reovirus to induce ER stress, we hypothesized that compounds targeting the UPR may sensitize cancer to reovirus. We identified that the PERK inhibitor, GSK2606414, can sensitize head and neck cancer cells to reovirus. GSK2606414 enhanced both reovirus protein production and reovirus-positive areas modeled in 3D spheroids. Profiling of UPR pathways revealed this increase correlated with elevated signaling through eIF2a and ATF4. This eIF2a-ATF4 signal was context dependent, occurring only due to the combination of reovirus and GSK2606414, not the canonical ER stress agent thapsigargin and GSK2606414. Use of short hairpin (shRNA) against eIF2a kinases revealed PERK or GCN2 as the kinases responsible for increased eIF2a-ATF4 pathway activity. Knockdown by shRNA confirmed ATF4 was necessary for the GSK2606414-induced increase in reovirus protein levels.

RESULTS

Inhibition of PERK Sensitizes HNSCC to Reovirus

The HPV-negative head and neck squamous cell carcinoma (HNSCC) cell lines HN5 (tongue) and FaDu (hypopharynx) were used; both are characterized by TP53 mutations and high EGFR and HER2 levels.[20] Survival experiments in 2D identified the PERK inhibitor GSK260414-sensitized FaDu and HN5 cells to a reovirus across a range of viral MOIs (Figure 1A). Values shown are corrected for drug-only toxicity. To assess drug-reovirus combination effects, we carried out Bliss independence analysis (Figure 1B). Greater than expected cell kill was observed when single-agent activity was compared with cell kill in combination. Reovirus MOIs of 20 and 75 were selected for FaDu and HN5 cells, respectively, because of these doses falling at the mid-point where combination efficacy was observed. These were used for all subsequent 2D and 3D assays.

The ability of GSK2606414 to increase the efficacy of reovirus was assessed in 3D tumor spheroids. 3D models were used to augment 2D assays because 3D models are both a more clinically relevant method to model stress and were viewed as an approach to modeling the area of viral infection. Fluorescent ubiquitination cell-cycle indicator-expressing[21] FaDu and HN5 cells were used to allow a more accurate assessment of spheroid area than bright-field images alone. Representative images after 7 days of GSK2606414 and reovirus infection are shown (Figure 1C). Spheroids were imaged over 11 days after the addition of GSK2606414 and reovirus. Automated image quantification of spheroid area based on fluorescence from multiple experiments is shown (Figure 1D). GSK2606414 enhanced the efficacy of reovirus as measured by a reduction in spheroid area. Bliss independence analysis showed greater than expected reduction in area because of combination treatment compared with single agents alone (Figure 1E). Efficacy *in vivo* was confirmed using both Tet-inducible PERK shRNA (shPERK) knockdown (Figure 1F) and GSK2606414 in combination with reovirus (Figure 1G). Tumor volume reduction by reovirus was significantly higher in the shPERK group compared with scrambled knockdown (shSCR) control and in combination with GSK2606414. Validation of PERK knockdown *in vivo*, efficacy of PERK knockdown in combination with reovirus *in vitro*, and *in vivo* curves in mm^3 are shown in Figure S1.

GSK2606414, but Not PERK Knockdown, Increases Reovirus Protein Levels *In Vitro* and *In Vivo*

After confirming increased reovirus efficacy in combination with GSK2606414, we investigated the impact of GSK2606414 on reovirus replication. Reovirus capsid protein levels were assessed in 2D culture by western blot (Figure 2A), and reovirus particle production by tissue culture infectious dose 50 ($TCID_{50}$) (Figure 2B). GSK2606414 increased the levels of capsid proteins σ3 and μ1C (Figure 2A). GSK2606414 also increased viable reovirus particle production by $TCID_{50}$, although this result was not statistically significant. It can be stated that viable reovirus particle production remains, at a minimum, undiminished despite decreasing cell viability in combination with GSK2606414.

To probe the effects of GSK2606414 in comparison with PERK knockdown, we used 3D tumor spheroids to model the area of reovirus infection. Tumors at the end of *in vivo* experiments in Figures 1F and 1G were also assessed for reovirus by fluorescence-based immunohistochemistry (IHF). Spheroids were treated with GSK2606414 and reovirus concurrently. After 96 h, spheroids were formalin fixed, paraffin embedded and sectioned. Sections were stained for σ3 and μ1C by fluorescence-based IHF and confocal images quantified by automated image analysis. An overview of the image analysis pipeline is shown (Figure 2C). Image segmentation was restricted to the peripheral edge of spheroids corresponding to a depth of 25 μm. This approach was taken due to localization of the majority of reovirus infection to the spheroid periphery. 3D spheroid sections indicated GSK2606414 enhanced the area that stained positive for reovirus infection as measured by μ1C (Figure 2D) and σ3 (Figure 2E). This could be attributed to an increase in the total number of infected cells because of GSK2606414, or an increase in reovirus capsid levels in cells at an early stage in infection compared with reovirus-only conditions. Tet-inducible knockdown was used as described for Figure 1. PERK knockdown by 96-h pre-treatment with doxycycline to induce scrambled or shPERK did not alter the percentage area positive for reovirus in 3D spheroids (Figure 2E). Quantification of reovirus-positive areas at days 18 and 20, respectively, from GSK2606414 or PERK knockdown *in vivo* experiments showed an increase because of GSK2606414, but not PERK knockdown, similar to observations *in vitro* (Figure 2F). These analyses indicated that although both PERK knockdown and GSK2606414 enhance tumor control by reovirus, only GSK2606414 quantifiably increased reovirus protein levels.

GSK2606414 Alters ER Chaperone Composition in Response to Reovirus

Reovirus has previously been shown to increase levels of ER-resident chaperones, such as GRP78 and protein disulphide isomerase

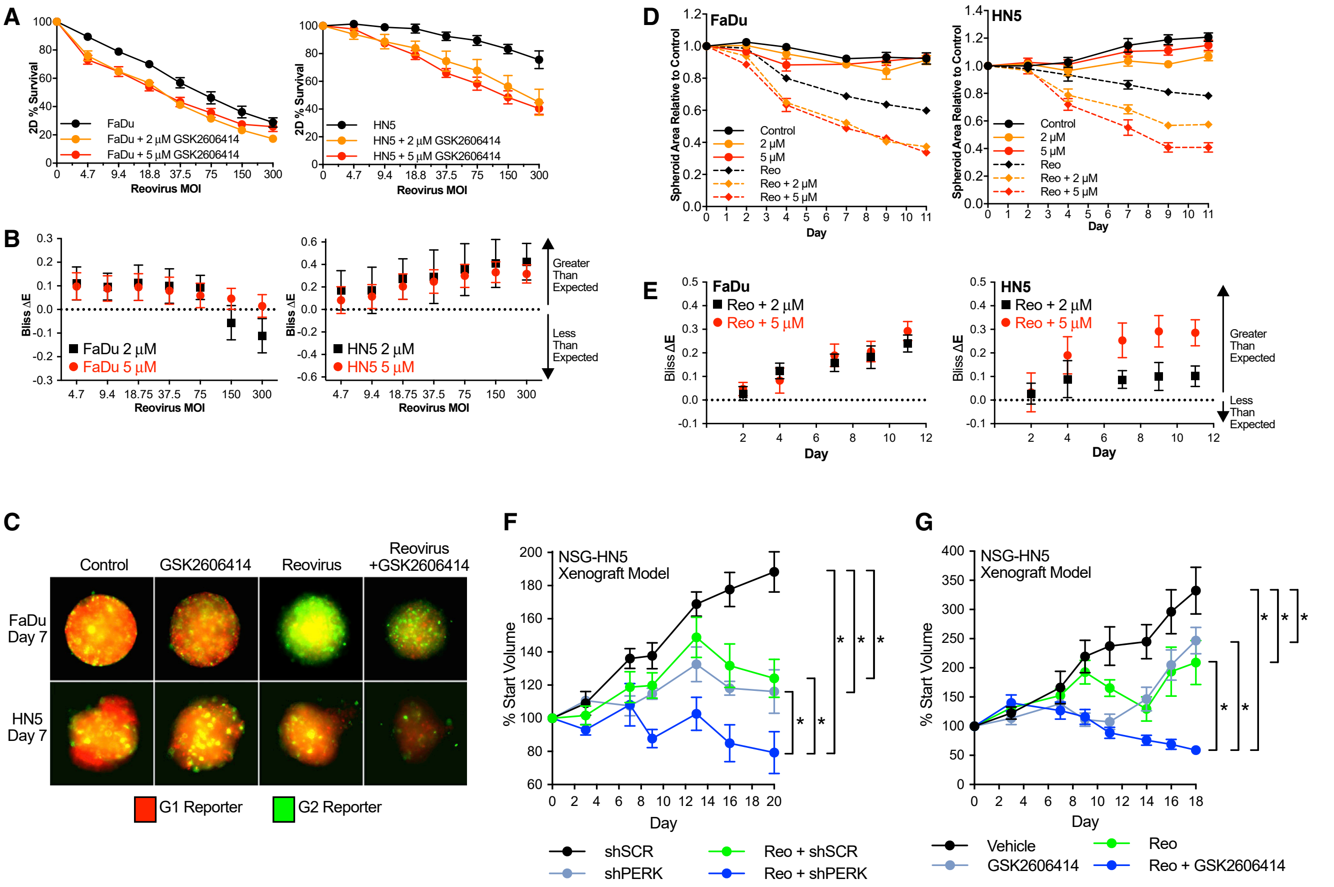

Figure 1. GSK2606414 Sensitizes HNSCC to Reovirus

(A) Cell viability of FaDu and HN5 HNSCC cell lines was determined at 72 h by MTT assay. GSK2606414 and reovirus were added concurrently. (B) Observed cell kill versus expected cell kill for MTT assays was determined by Bliss independence analysis. Expected cell kill is calculated on the assumption that single agents are non-interacting. Positive ΔE corresponds to greater observed kill than expected, and negative ΔE less than expected. Full details are outlined in Materials and Methods. (C) FaDu and HN5 HNSCC cell lines were plated in low-attachment U-bottom plates to form 3D tumor spheroids expressing G1-mCherry and G2-AMCyan cell-cycle trackers. Spheroids were treated with GSK2606414 and reovirus concurrently. Spheroids were imaged using cell-cycle tracker fluorescence at the indicated time points. (D) Area relative to control was calculated by automated image quantification using images as shown in panel C. (E) Observed reduction versus expected reduction for 3D spheroid areas was determined by Bliss independence analysis as in (B). (F) Increased efficacy of reovirus in combination with loss of PERK was determined *in vivo* by Tet-inducible shRNA. HN5 cells stably expressing lentiviral Tet-pLKO-puro were injected subcutaneously into NSG mice. HN5 cells contained either scrambled shRNA (SCRsh) or PERK targeting shRNA (PERKsh). All mice received 50 mg/kg doxycycline daily by gavage with reovirus delivered by intra-tumoral injection at day zero. Tumor volumes are expressed relative to start volume. For validation of knockdown, see Figure S1. (G) Increased efficacy of reovirus in combination with GSK2606414 was determined *in vivo* in HN5 cells injected subcutaneously into NSG mice. Mice received 50 mg/kg GSK2606414 on days 0–4, 7–11, and 14–18 with two injections of reovirus on days 0 and 9. (A and D) Data are ±SEM of at least three independent experiments. Bliss independence analysis in (B) and (E) shown with 95% confidence intervals. *In vivo* statistical analysis shown between groups in (F) and (G) by unpaired t test, *p < 0.05 of area under curve comparison for individual tumors.

(PDI).[16] We sought to assess how GSK2606414 may modulate alterations in ER chaperone levels caused by reovirus infection using the same 3D tumor spheroid approach used to model reovirus infection *in vitro* (Figure 3). As in Figure 2, spheroids were treated with reovirus and GSK2606414 for 96 h before formalin-fixed paraffin-embedded (FFPE) processing, sectioning, and IHF imaging by confocal microscopy. Automated image quantification was used to quantify areas of high chaperone expression as outlined for Figures 2C–2E. This was isolated to the spheroid periphery as described previously for reovirus infection. In addition, the core of HN5 spheroids displayed high levels of ER chaperones, and peripheral quantification excluded changes in this core region not directly linked to reovirus infection (shown in image inset in Figure 3A).

Representative images are shown for the chaperone GRP78 (Figure 3A), the ER retention motif KDEL (Figure 3B), and the chaperone PDI (Figure 3C). GRP78 levels significantly increased following reovirus infection in both cell lines (Figure 3D). GSK2606414 inhibited GRP78 induction at the edge of spheroids in both cell lines across all doses tested. The ER retention motif,

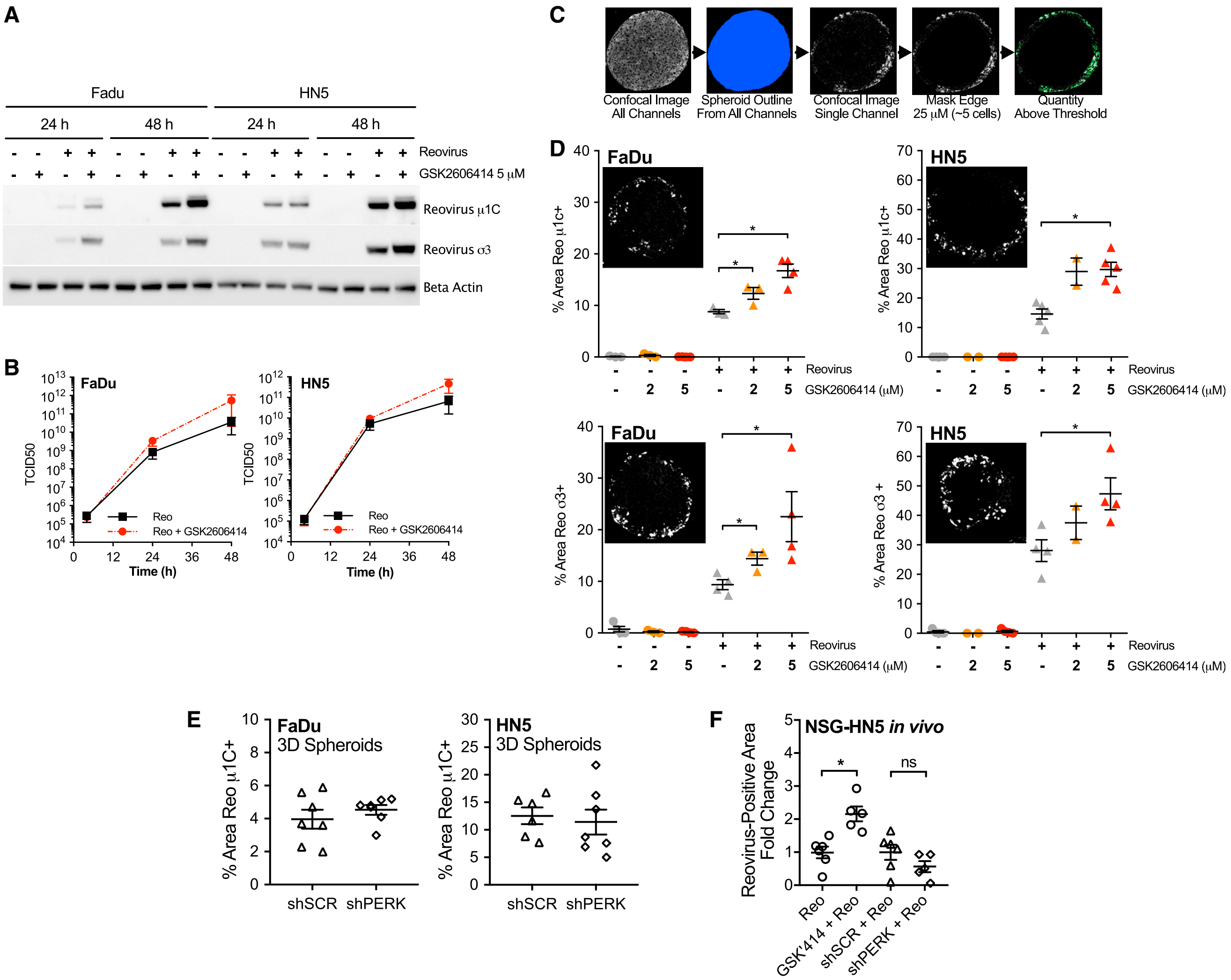

Figure 2. GSK2606414 Increases Reovirus Infection in 2D and Modeled in 3D Tumor Spheroids

(A) Lysates from 2D cell culture at the time points indicated after treatment were probed by western blot for the reovirus capsid proteins μ1C and σ3. (B) Viable replicating reovirus particle production from media and cells combined from 2D culture was quantified by $TCID_{50}$ assay. (C) HNSCC cell lines were plated in low-attachment U-bottom plates to form spheroids and treated as in Figure 1. 96 h after treatment, spheroids were collected, formalin fixed, paraffin embedded, and sectioned. Sections were stained for reovirus μ1C and σ3 by IHF to quantify the area positive for reovirus infection. Due to reovirus accessing and infecting only the peripheral edge of the spheroid as shown, quantification was restricted to a depth of 25 μM. Further details on this rationale are outlined in the Results. (D) Confocal images were quantified by automated image quantification of infected areas as illustrated. ×20 field of view shown corresponds to 415 × 415 μm. (E) 3D spheroids containing SCR or shPERK were treated with doxycycline for 96 h before infection with reovirus. After a further 96 h in doxycycline, spheroids were collected and stained for reovirus μ1C as described for (C) and (D). (F) FFPE tumors from the end of the experiment from Figures 1F and 1G were sectioned and stained for reovirus μ1C by IHF. Slides were imaged on a Perkin Elmer Vectra 3, and the percentage area positive for reovirus quantified using cell profiler software was averaged from two sections. Reovirus-positive areas are expressed as fold change relative to reovirus alone for GSK2606414, or scrambled shRNA for PERK shRNA knockdown. Spheroid data points represent the average of four to eight spheroids within a single independent experiment. All figure data are ±SEM; statistical analysis by unpaired t test, $^{*}p < 0.05$.

KDEL, was stained to quantify global ER-resident protein levels (Figure 3E). Reovirus induced an increase in global ER-resident proteins measured by KDEL-high areas that was further increased in combination with GSK2606414. Similar to KDEL, PDI-high levels increased because of reovirus infection and were further increased by GSK2606414 addition (Figure 3F). When comparing reovirus capsid staining patterns in Figure 2 with GRP78, KDEL, and PDI staining, a degree of co-localization was apparent only for PDI and capsid proteins in HN5 cells. Staining of GRP78 and KDEL, particularly in FaDu spheroids, was diffuse relative to highly discrete reovirus capsid-positive areas. This indicates that stress induced by reovirus may be caused by a combination of a bystander effect or also occur at early stages of infection where capsid proteins are not yet detectable.

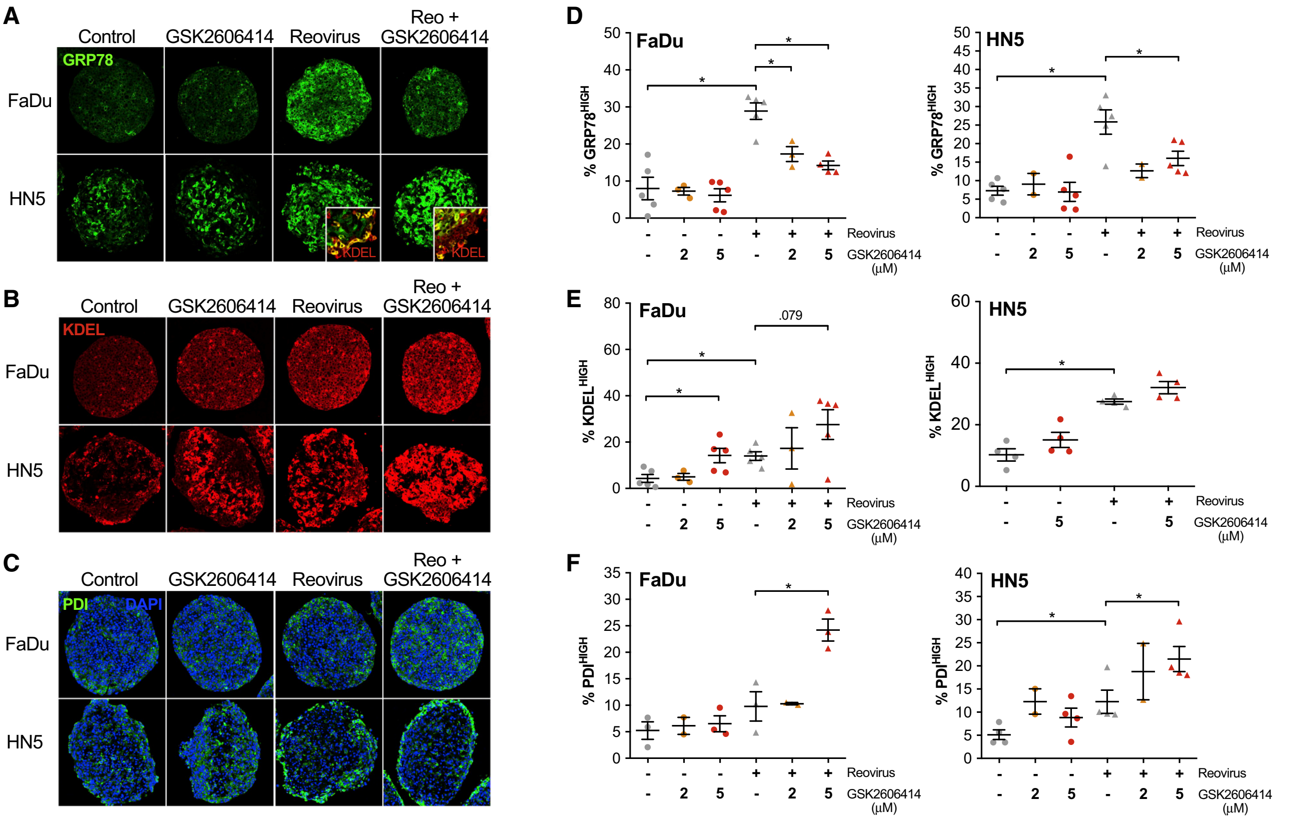

Figure 3. GSK2606414 Inhibits Reovirus-Induced GRP78 while Increasing PDI and Overall ER Resident KDEL Levels

FaDu and HN5 HNSCC 3D tumor spheroids were treated with GSK2606414 and reovirus for 96 h before spheroids were formalin fixed, paraffin embedded, and sectioned. Sections were stained by IHF for (A) GRP78, (B) KDEL, and (C) PDI. The ×20 field of view shown corresponds to 415 × 415 μm. Confocal images were quantified for areas of high expression and restricted to the peripheral 25 μM as previously outlined in Figure 2. Quantification for both FaDu and HN5 cells shown for (D) GRP78, (E) KDEL, and (F) PDI. Each point represents an independent experiment where each experiment is an average of four to eight spheroids. Data are ±SEM with statistical analysis by unpaired t test, *$p < 0.05$.

Analyses mirroring spheroid data were performed on *in vivo* samples from the end of the experiment at days 18 and 20 (Figure S2). GSK2606414 in combination with reovirus *in vivo* strongly mirrored the increase in PDI observed in spheroids, with decreased GRP78 less clear. PERK knockdown did not alter PDI levels because of reovirus, but increased GRP78 alone at an earlier day 12 time point or at day 20 in reovirus-infected areas. These data indicate that both PERK knockdown and GSK2606414 can aggravate/alter the ER stress response to reovirus, but the resulting profiles, as measured by the GRP78 and the redox chaperone PDI, are clearly divergent.

GSK2606414 Reduces Signaling through XBP1 and ATF6 while Increasing Signaling via eIF2a-ATF4

To understand what may be behind this perturbation of ER chaperones, we generated reporter constructs to investigate the three UPR signaling pathways responsible for regulating chaperone levels. An IRE1a endonuclease reporter contained the 26-nt intron from XBP1 in front of GFP. Splicing and removal of this intron, as is the case with XBP1 to spliced XBP1, placed GFP in frame (IRE1alpha endonuclease reporter). Downstream of PERK and eIF2a, the 5′ upstream regulatory sequence of ATF4 was cloned in front of the start codon of mCherry (ATF4 promoter). Multiple repeats of the previously published ATF6 transcription factor binding site[22] were cloned in front of mCherry (ATF6 reporter). All three are illustrated (Figure 4A). The canonical ER stress-inducing agent, thapsigargin, was used to validate UPR reporter signaling in combination with GSK2606414 (Figure 4B). Activation of all UPR reporters by thapsigargin was observed, except for IRE1 reporter activity in FaDu cells. Inhibition of thapsigargin-induced ATF4 reporter levels was clearly observed for GSK2606414, with compensatory increases in other UPR signal pathways (except IRE1 in FaDu cells).

After reporter validation, ATF4, IRE1, and ATF6 reporter levels were imaged after 72 h of reovirus infection and GSK2606414 treatment. Representative images are shown (Figure 4C). Automated image quantification was used to determine reporter intensity across multiple independent experiments (Figure 4D).

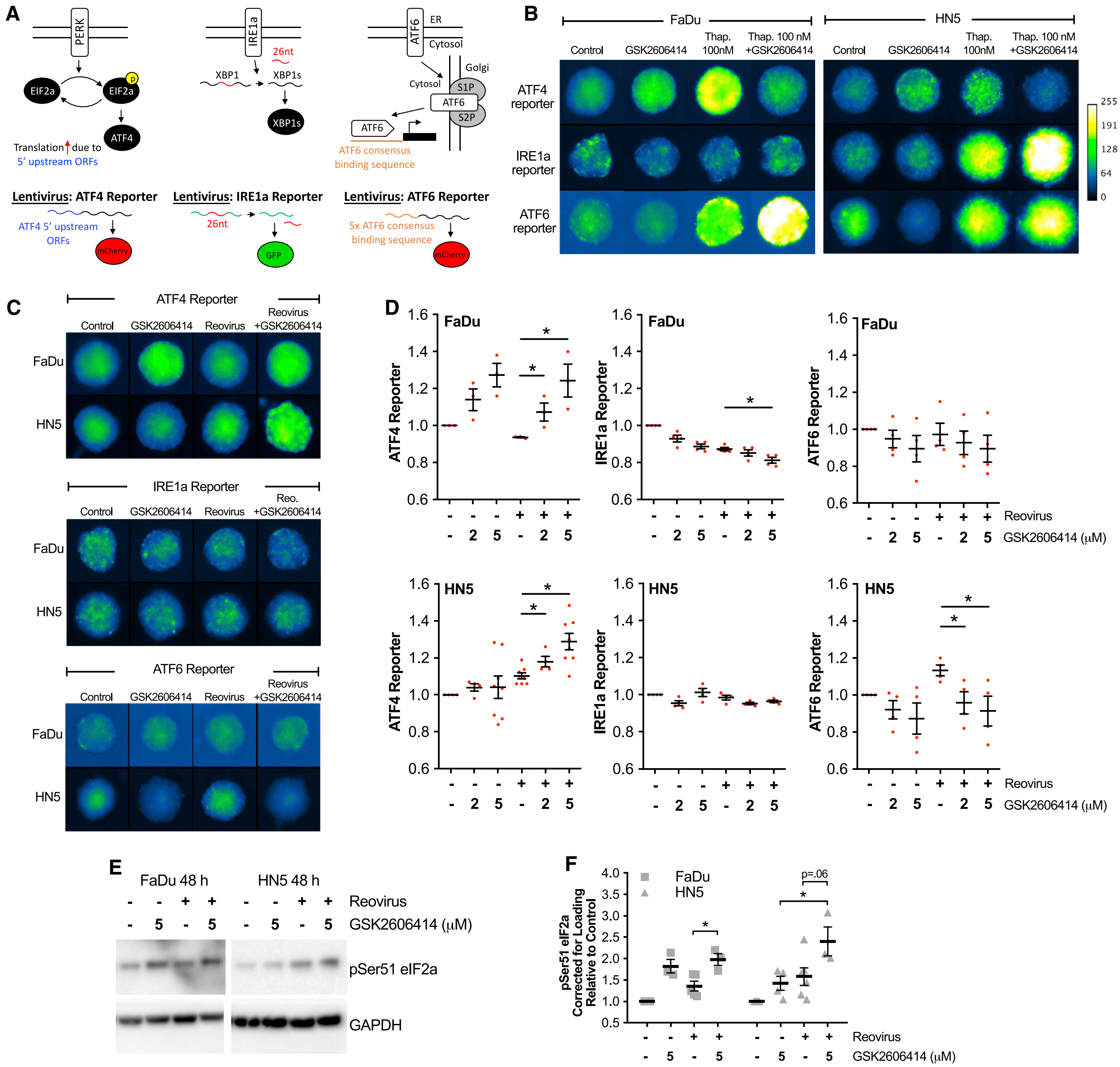

Figure 4. GSK2606414 Increases UPR Signaling through eIF2a-ATF4 while Decreasing Signaling through IRE1alpha and ATF6

(A) Illustration of tripartite UPR reporter design for live-cell imaging of IRE1a splicing of XBP1 intron sequence, ATF4 translation downstream of eIF2a, and activity of the ATF6 consensus DNA binding sequence. (B) 3D tumor spheroids containing reporter constructs were treated with 100 nM thapsigargin and 5 μM GSK2606414, and imaged at 72 h to profile lentiviral UPR reporter constructs. To aid clarity, grayscale images are presented using the pseudo-color scale shown. (C) 3D tumor spheroids containing reporter constructs were treated with reovirus and GSK2606414, and imaged at 72 h. (D) Automated image analysis was used to identify spheroids and calculate average reporter intensity. Each data point represents an independent experiment, each containing the average of at least four spheroids. (E) 2D cell lysates were collected after 48-h treatment of cells with reovirus and GSK2606414. Lysates were probed for pSer51 EIF2a. (F) Densitometry quantification of pSer51 eIF2a from western blots of three independent experiments. Data are corrected for loading and normalized to control. Data are ±SEM of a minimum of three independent experiments with statistical analysis by unpaired t test, *p < 0.05.

No activation of the three arms of the UPR was observed because of reovirus in FaDu spheroids, whereas ATF6 and ATF4 activity was observed in HN5 spheroids, although some degree of activation of ATF4 by reovirus was observed in later experiments (see Figures 5B–5E). The most pronounced difference was an increase in ATF4 reporter levels due to GSK2606414 alone. This was highly

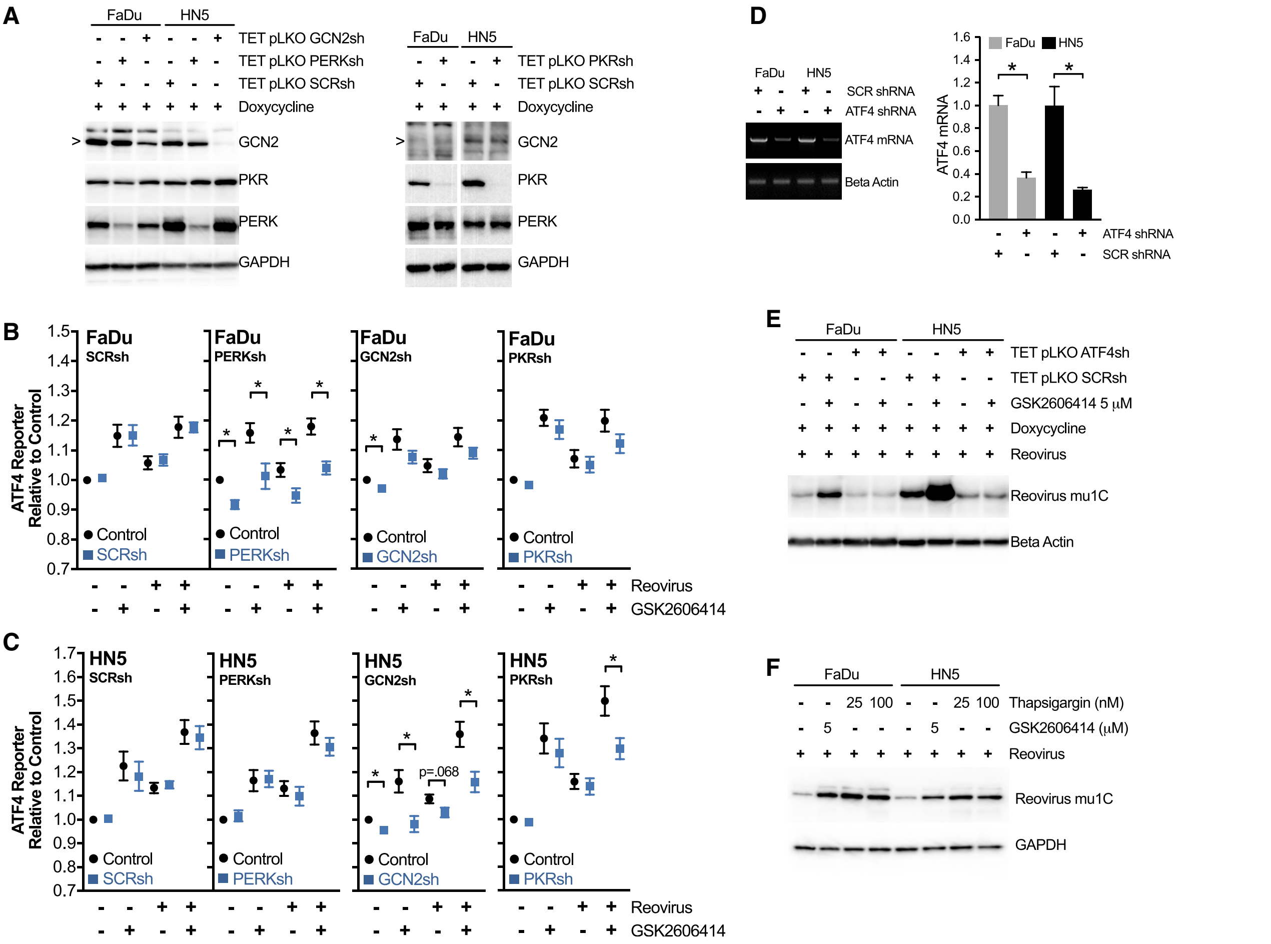

Figure 5. In the Context of Reovirus Infection, GSK2606414 Promotes eIF2a-ATF4 Signaling via PERK or GCN2 with Increased Viral Protein Levels ATF4 Dependent

(A) Western blot of cell lysates from 2D culture validating Tet-pLKO shRNA knockdown of PERK, GCN2, and PKR after 96 h of doxycycline treatment. (B) FaDu and (C) HN5 cells were co-infected with ATF4-mCherry reporter in combination with Tet-pLKO SCR, PERK, GCN2, or PKR targeting shRNA. Spheroids were treated for 96 h with doxycycline before treatment with GSK2606414 and reovirus. Reporter expression was imaged at 72 h, matching the time point used in Figure 4. Control values (black circles) are for parental Tet-pLKO cell lines in the absence of doxycycline knockdown, and shRNA values (blue squares) correspond to doxycycline-induced shRNA expression and knockdown. (D) FaDu and HN5 cells were infected with Tet-pLKO-inducible shRNA targeting ATF4 or scrambled control. ATF4 transcript variant 2, but not variant 1, was present in both cell lines. Knockdown after 96 h of doxycycline treatment was confirmed by PCR. Quantitation of PCR band intensity from three independent experiments expressed relative to SCR control for each cell line. (E) After 96 h of pre-treatment with doxycycline to establish ATF4 knockdown, cells were exposed to reovirus and GSK2606414 for 48 h before analysis of reovirus μ1C protein levels by western blot. (F) Cell were treated with DMSO control, GSK2606414, or thapsigargin in combination with reovirus and analyzed at 48 h for μ1C protein levels by western blot. Data are ±SEM of a minimum of three independent experiments with statistical analysis by unpaired t test, *p < 0.05.

pronounced in FaDu cells. ATF4 reporter levels increased further due to the combination of reovirus and GSK2606414 in HN5 cells alone. To validate signaling upstream of ATF4, levels of pSer51 eIF2a were determined from 2D cell lysates by western blot (Figure 4E). Densitometry from at least three independent blots is also shown (Figure 4F). The pattern of pSer51 eIF2a was similar to that observed with the ATF4 reporter. These data indicated that signaling through eIF2a-ATF4 appeared to be the main mechanistic change in UPR signaling during exposure to GSK2606414 and reovirus infection.

Increased Reovirus Protein Levels Due to GSK2606414 Are ATF4 Dependent and Due to Increased Signaling via PERK and GCN2

In the comparison between thapsigargin and reovirus in Figure 4, we noted that the effect of GSK2606414 was context dependent. Thapsigargin is a much stronger activator of UPR signaling than reovirus. In this context, GSK2606414 strongly inhibits ATF4 protomer activity. However, in the thapsigargin experiment (Figure 4B), as well as with reovirus (Figure 4C), we noted GSK2606414 alone caused an increase in ATF4 reporter signaling. Western blot data (Figure 4F) indicated that upstream pSer51 eIF2a levels correlated to ATF4 reporter levels.

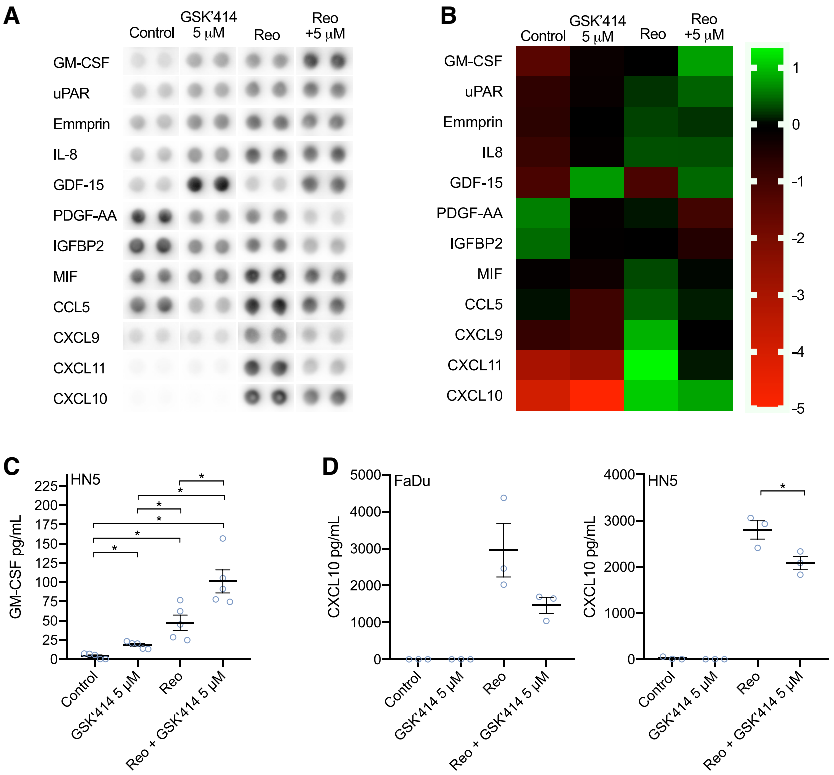

Figure 6. GSK2606414 Modulates Cytokine Production in Response to Reovirus, Increasing GM-CSF Levels

A human cytokine array was used to assess cytokine secretion *in vitro* in response to reovirus in combination with GSK2606414 after 48 h. Only cytokines where changes were observed are shown (A), with levels quantified and displayed also by heatmap (B). Findings for (C) GM-CSF and (D) CXCL10 were validated by ELISA using media at 48 h. Data are ±SEM of a minimum of three independent experiments with statistical analysis by unpaired t test, *$p < 0.05$.

Based on previous data relating to reovirus and the ISR,[23] we believed that this may be responsible for the increase in reovirus protein production and infection. To help elucidate the mechanism behind this, we used doxycycline-inducible shRNA against the ISR eIF2a kinases, PERK, GCN2, and PKR (shRNA validation; Figure 5A).

The ATF4 reporter was combined with scrambled, PERK, GCN2, or PKR shRNA, and the experiments of Figure 4 were repeated. Knockdown of each eIF2a kinase was used to determine the source of ATF4 reporter activity (Figures 5B and 5C). All four shRNA variants were tested in the absence of doxycycline (black circles) and shown to have similar results to earlier non-shRNA cell lines in Figure 4. Induction of scrambled shRNA with doxycycline (blue squares) was shown to have no effect on ATF4 reporter activity due to GSK2606414 and/or reovirus compared with no doxycycline control conditions. Doxycycline-induced knockdown of PERK reduced ATF4 reporter activity in all conditions in FaDu cells. This was most pronounced in GSK2606414 treatment conditions. No reduction was observed in HN5 cells. Doxycycline-induced knockdown of GCN2 was seen to reduce ATF4 reporter activity in all conditions in HN5 cells. Although a small decrease was seen in FaDu cells, this was not statistically significant. Doxycycline-induced knockdown of PKR significantly reduced the ATF4 reporter signal in HN5 cells treated with the combination of GSK2606414 and reovirus.

In the context of previous comparative data between PERK knockdown and GSK2606414 (Figure 2; Figure S2), it is clear that PERK knockdown alone does not increase ATF4 reporter activity in either FaDu or HN5 cells (Figures 5B and 5C). This was a mechanistically distinct difference between PERK knockdown and GSK26006414, and potentially the reason behind the disparate effects observed when assessing reovirus levels in Figure 2.

To test whether increasing pSer51 eIF2a levels (Figures 4E and 4F) and ATF4 reporter levels (Figures 4D and 5B–5E) due to GSK2606414 was the key driver behind increased reovirus protein levels, we used doxycycline-induced knockdown of ATF4 (ATF4sh). Knockdown was validated at the mRNA level by PCR (Figure 5D). Transcript variant 2 was detectable in both cells, with mRNA levels significantly reduced with doxycycline treatment. ATF4sh blocked increased reovirus μ1C levels because of GSK2606414 (Figure 5E). Unlike in combination with thapsigargin, in the context of reovirus infection, GSK2606414 can increase signaling through eIF2a-ATF4. ATF4sh confirmed this signaling event as the reason behind increased reovirus protein replication due to GSK2606414. As an additional confirmation, we tested whether the potent induction of ATF4 reporter activity observed in Figure 4B by thapsigargin alone could also increase reovirus levels. This proved to be the case (Figure 5F), suggesting conditions that enhance ATF4 activity have positive consequences for reovirus protein production.

GSK2606414 Increases GM-CSF Secretion in Combination with Reovirus

To assess the impact of GSK2606414 on reovirus-induced cytokine secretion, we used an array to profile alterations to cytokine secretion *in vitro* in HN5 cells (Figures 6A and 6B). This indicated changes to a number of cytokines. Most prominent were increased levels of granulocyte-macrophage colony-stimulating factor (GM-CSF) and decreased levels of a number of T cell chemoattractants, such as CXCL9, CXCL10, and CXCL11, as well as decreased CCL5. GM-CSF and CXCL10 findings were validated by ELISA in both FaDu and HN5 cells. This confirmed the increase of GM-CSF in combination with reovirus in HN5s (Figure 6C). FaDu cells did not appear to secrete GM-CSF under any conditions, in keeping with other data on radiation that also showed an absence of GM-CSF expression in FaDu

cells.[24] A decrease in reovirus-induced CXCL10 secretion by GSK2606414 was observed for both FaDu and HN5 cells. It should be noted, however, that GSK2606414 reduced CXCL10, but the levels in the combination group were still substantially greater than untreated controls. These data indicate that GSK2606414 may aid dendritic cell infiltration and antigen presentation through GM-CSF, but potentially dampen T cell chemotaxis through reduced levels of the CXCR3 ligands CXCL9–11.

DISCUSSION

Previous publications have shown that inhibition of BRAF or MEK enhanced the efficacy of reovirus via a mechanism involving ER stress.[17] We hypothesized that direct targeting of UPR signaling may present an opportunity to sensitize HNSCC to reovirus. This led us to identify the PERK inhibitor, GSK2606414, as increasing the efficacy of reovirus in HNSCC.

We observed that GSK2606414 increases reovirus protein levels measured by western blot, as well as the area positive for infection in 3D spheroids and *in vivo*. Tripartite UPR signaling reporters were created to allow monitoring of the UPR in 3D. Initial validation with GSK2606414 and thapsigargin was as expected, with ATF4prom activity blocked by GSK2606414. This coincided with a compensatory increase in the IRE1a and ATF6 UPR pathways. Our original expectation was that reovirus would act much the same as thapsigargin. However, this was not the case, with clear differences in UPR signaling due to reovirus compared with thapsigargin. Whereas GSK2606414 blocked canonical thapsigargin-induced eIF2a-ATF4 signaling, single-agent GSK2606414 yielded an increase. This increase was maintained in FaDu cells and further enhanced in HN5 cells following co-treatment with reovirus. The effect of GSK2606414 was, therefore, context dependent. In a search of the literature, we were able to corroborate these findings. Single-agent GSK2606414 has been shown previously to increase pSer51 eIF2a,[25] although this specific observation was not discussed in that study. To our knowledge, this is the first commentary on this observed phenotype in the literature.

In vivo and *in vitro* data indicated PERK knockdown or GSK2606414 increased tumor control by reovirus, yet the profile of ER stress markers differed. PERK knockdown clearly increased GRP78 levels at day 12 *in vivo* and increased GRP78 levels in reovirus-infected areas, as well as adjacent areas likely to be in the early stages of infection. Upregulation of GRP78 is a marker of UPR induction and elevated stress. This aggravated stress appears mechanistically different from GSK2606414. Yet either aggravation or perturbation of ER stress signaling by PERK knockdown or GSK260414, respectively, appears to share the same therapeutically beneficial outcome. PERK knockdown failed observably to increase reovirus levels as shown for GSK2606414. These data are in keeping with the identified ATF4-dependent mechanism because PERK knockdown does not induce ATF4 reporter signaling. The precise contribution of these quantifiably increased reovirus metrics to efficacy is difficult to determine. It is logical to conclude that conditions whereby anti-tumor efficacy coincides with increased reovirus persistence is anticipated to be a more therapeutically beneficial scenario.

Activation of the ISR has been associated with a cellular environment supportive of reovirus replication.[23] Isogenic pairs of murine embryonic fibroblasts (MEFs) rather than cancer cell lines were studied, but a possible role in therapeutic efficacy was not investigated. In that study, reovirus replication was not altered in PERK knockout MEFs but was reduced by ATF4 knockout. Our data in a therapeutic context agree with these findings. Increased eIF2a-ATF4 signaling activity due to GSK2606414 corresponded to increased reovirus protein levels, with ATF4 knockdown abolishing this increase.

To determine the mechanism behind the increase in eIF2a-ATF4 activity due to GSK2606414, we used shRNAs against the eIF2a kinases PERK, GCN2, and PKR in combination with an ATF4 reporter (Figures 5B and 5C). In FaDu cells, ATF4 reporter activity triggered by GSK2606414 was PERK dependent, whereas in HN5 cells, enhanced reporter activity was GCN2 dependent across all conditions, with PKR contributing only when reovirus was combined with GSK2606414. PERK, GCN2, and PKR all form dimers upon activation.[26–28] A recent publication has shown that PERK interacts with the actin regulator FLNA, independent of UPR signaling.[29] In that study, PERK null cells were reconstituted with PERK lacking the luminal domain. This clearly showed the ability of GSK2606414 to induce PERK dimerization independent of the luminal domain and without the need for thapsigargin treatment. The interaction of PERK-FLNA increased on treatment with GSK2606414, indicating that PERK dimerization and not just kinase activity is sufficient to drive this protein-protein interaction. It is plausible that in the context of reovirus infection, a similar mechanism of action may contribute to the phenotype we observed in this study.

The initial discovery that PERK inhibitors stabilized dimerization was made in the context of a structural similarity to RAF inhibitor-driven kinase dimerization.[30] Inhibitors of wild-type BRAF can drive activation of mutant RAS independently of kinase activity.[31–33] RAF can form homodimers or heterodimers with drug binding inhibiting one subunit of the dimer but inducing transactivation of the other.[33] In Lavoie et al.,[30] they concluded that PERK inhibition may result in transactivation of structurally similar GCN2, PKR, or HRI, although eIF2a kinase heterodimers have not been reported in the literature. The PERK-dependent increase in ATF4 activity due to GSK2606414 observed in FaDu cells would be in keeping with such transactivation[30] and dimerization events.[29] This suggests a mode of action where GSK2606414 in the context of reovirus infection enhances eIF2a-ATF4 signaling supporting enhanced viral protein production.

The lack of PERK dependence in HN5 cells does not fit this model, but due to the structural similarity of GCN2 to PERK, it is not inconceivable that some degree of off-target transactivation of GCN2 could be induced by GSK2606414 at the concentrations used. That this was not also observed in FaDu cells may be due to sub-optimal

knockdown of GCN2 because a non-significant decrease in GSK2606414 conditions was observed. It has been shown that GSK2606414 has off-target inhibitory effects, notably on RIPK1[34] and cKIT.[35] In experiments comparing RIPK1 inhibitors with GSK2606414 and thapsigargin (Figure S3), increased reovirus protein levels due to GSK206414 did not appear to be linked to off-target RIPK1 inhibitory effects. Knockdown data on PERK, GCN2, and ATF4 suggest that the effects observed in this study are isolated to UPR signaling alone.

In conclusion, our study reveals that GSK2606414 modulates UPR signaling in combination with reovirus in a fashion that is mechanistically different from the canonical ER stress-inducing agent thapsigargin. The context-dependent modulation of UPR signaling due to GSK2606414 allows increased translation of reovirus proteins in an ATF4-dependent manner. This can lead to an increase in the area positive for reovirus infection as modeled in 3D spheroids and *in vivo*. This suggests that future clinical translation should investigate the role of ER stress and in particular ATF4 in profiling susceptibility to reovirus. Combinations of reovirus and agents that enhance ER stress signaling through ATF4 should be considered for future clinical studies.

MATERIALS AND METHODS

Cell Culture and Compounds

FaDu and 293T cells were purchased from ATCC. LON-LICR-HN5 cell lines were from Prof. Sue Eccles (ICR, London, UK). Cells were cultured in DMEM, 5% FBS, 1% (v/v) glutamine, and 0.5% (v/v) penicillin/streptomycin. Short tandem repeat (STR) profiling was carried out by Bio-Synthesis. Mycoplasma testing used the e-Myco PCR kit (Intron Biotechnology). Experiments were carried out within 3 months of resuscitation. GSK2606414 was obtained from MedKoo Biosciences (NC, USA). Doxycycline hyclate was obtained from Sigma-Aldrich (Gillingham, UK). Reovirus type 3 Dearing (Reolysin/pelareorep) was kindly supplied by Oncolytics Biotech (Calgary, Canada).

MTT Viability Assay

Cells were seeded in 96-well plates. After 24 h, GSK2606414 and reovirus were added concurrently. Viability was determined at 72 h by MTT assay. Absorbance at 550 nm was measured and viability normalized to control DMSO-treated cells.

3D Spheroid Size Imaging

Cells were plated in ultra-low-attachment plates (#7007; Corning). Cells contained cell-cycle tracker proteins as previously described,[21,36] which were used to visualize spheroid area. Medium was refreshed every 48 h. At 96 h post-plating, GSK2606414 and reovirus were added. GSK2606414 was refreshed every 48 h after addition. Spheroids were imaged at a fixed exposure throughout the experiment on an EVOS FL microscope (Thermo Fisher, UK). Spheroid area was quantified by automated image quantification of mCherry and AMCyan channels combined using Cell Profiler v.3 (Broad Institute, MA, USA).

Immunoblotting

Cells were scraped in radioimmunoprecipitation assay (RIPA) buffer (Thermo Fisher) containing 2 mmol/L Na_3VO_4 and protease inhibitors. Supernatants were quantified by BCA assay (Pierce), separated by SDS-PAGE, transferred to polyvinylidene fluoride (PVDF) membrane (Thermo Fisher), and blocked in Tris-buffered saline (TBS) with 5% non-fat dry milk and 0.1% Tween 20. Membranes were probed with the antibodies: pSer51 eIF2a #3398, PERK #5683, GCN2 #3202, PKR #12297, and GAPDH #2118 from Cell Signaling Technology (MA, USA); reovirus μ1C 10F6 and reovirus σ3 4F2 were from the Developmental Studies Hybridoma Bank (IA, USA); and ATF4 ab184909 and Beta Actin ab8226 were from Abcam (Cambridge, UK).

One-Step Viral Growth $TCID_{50}$ Assay

Cells were plated in 24-well plates. After 24 h, cells were infected with reovirus with or without GSK2606414. After 2 h, media were removed and cells were washed once with PBS. Fresh reovirus free media with or without GSK2606414 was added. Cells and supernatant were harvested and freeze-thawed three times. Resulting supernatants were titered by serial dilution on L929 cells as previously described.[37]

Immunofluorescence

Spheroids were rinsed in PBS before fixation in 10% neutral-buffered formalin (NBF). Spheroids were embedded in HistoGel before paraffin embedding and sectioning. *In vivo* tumors were fixed in NBF before paraffin embedding and sectioning. Heat-induced antigen retrieval was performed using pH 6 sodium citrate buffer. Tissue was blocked using 5% BSA before incubation with the following antibodies: PERK #5683, GRP78 #3177, and PDI #3501 were from Cell Signaling Technologies (MA, USA); KDEL 10C3 was from Enzo (Exeter, UK); and reovirus μ1C 10F6 and reovirus σ3 4F2 were from the Developmental Studies Hybridoma Bank (IA, USA). Alexa Fluor 488, 546, and 647 conjugates of anti-rabbit IgG H+L, anti-mouse IgG H+L, anti-mouse IgG2a, and anti-mouse IgG2b were from Thermo Fisher (UK). Spheroid sections were imaged on a Zeiss 710 confocal (Jena, Germany) with quantification using Cell Profiler v.3. Exposure settings were based on the minimum possible, which still allowed robust spheroid identification for segmentation. Controls were compared during exposure setup to ensure similar intensities across experiments. Stained sections from *in vivo* tumor samples were imaged on a Perkin Elmer Vectra 3.0 with spectral unmixing of images performed using Perkin Elmer Inform software. Image quantification was performed using Cell Profiler v.3 as described in the Results.

Cytokine Profiling

Media 48 h after treatment with reovirus and GSK2606414 were collected and centrifuged to remove cells or debris. Initial profiling used the proteome profiler human XL cytokine array kit from R&D Systems (MN, USA). Array results were quantified by densitometry using ImageJ (FIJI v.2). Validation of array findings used the human CXCL10/IP-10 DuoSet and human GM-CSF DuoSet ELISA kits from R&D Systems.

UPR Reporter Constructs and High-Content Imaging

Reporter constructs were cloned into the lentivirus pHRSIN (kindly provided by Prof. Greg Towers, University College London [UCL], London, UK). Primers are listed in Table S1. The region encoding the 26-nt intron from XBP1 excised by IRE1a riboendonuclease activity was incorporated by PCR in front of EGFP-FLAG and inserted between SFFV and WPRE elements (LV-IRE1endo). 1,800 bp upstream of the ATF4 start codon was amplified from genomic DNA by PCR, with a second PCR referred to as extended primers adding overlap assembly regions at the 5′ end for pHRSIN and the 3′ end for mCherry. mCherry was amplified by PCR using an initial PCR including a previously cloned C-terminal MYC tag, followed by a second PCR to add a 3′ overlap assembly region with pHRSIN. This was assembled into pHRSIN using NEBuilder (NEB, USA), resulting in mCherry with the 5′ 1,800-bp genomic sequence of ATF4 and a 3′ WPRE element from pHRSIN (LV-ATF4prom). Using the previously published ATF6 binding site,[22] we inserted 5-repeats of the binding site (ID 11976; Addgene) in front of EGFP-WPRE (LV-ATF6bind). Lentiviral reporter constructs were packaged using MD2.G and psPAX2 in 293T cells using Lipofectamine 2000. Supernatants containing polybrene were used to infect cell lines with reporter constructs individually and selected using puromycin or blasticidin. Reporter expression was measured using a Celigo S high-content imaging system from Nexcelom Bioscience (MA, USA). Spheroid recognition and average reporter intensity were quantified using Cell Profiler v.3.

Tet-pLKO-puro shRNA

shRNA sequences for scrambled, PERK, GCN2, PKR, and ATF4 were cloned into the Tet-pLKO-puro system.[38] Target sequences were: scrambled, 5′-GACAAGTTAAGAACCGCGA-3′; PERK, 5′-CCGTGAAAGCATGGAAACA-3′; GCN2, 5′-TGGCTAAGCAGGAACGTTT-3′; PKR, 5′-GGGCTAATTCTTGCTGAAC-3′; and ATF4, 5′-TGCTTACGTTGCCATGATC-3′. Knockdown was validated after 96 h in 400 μg/mL doxycycline with fresh doxycycline added at 48 h. In all experiments, doxycycline was refreshed every 48 h until the end of the experiment. GSK2606414 and reovirus addition took place after 96 h of pre-treatment with doxycycline. Validation was done by western blot, except for ATF4, which was by PCR. For PCR validation, RNA was prepped using an RNeasy Plus kit (QIAGEN), converted to cDNA using ProtoScript II (NEB), with PCR performed using GoTaq DNA polymerase (Promega). ATF4 transcript variant 1 and 2 primers are listed in Table S2. In studies combining Tet-pLKO-puro shRNA with LV-ATF4prom, puromycin and blasticidin were used sequentially for selection of positively infected cells. Imaging and quantitation were performed as outlined previously for UPR reporters.

In Vivo

All experiments were approved by the institutional ethics review board. Female NOD scid gamma (NSG) mice were obtained from Charles River, and 2 million HN5 cells were implanted subcutaneously. HN5 cells were parental or contained Tet-pLKO-puro SCR shRNA or PERK-targeting shRNA. Scrambled shRNA groups contained n = 6 mice and n = 5 shPERK. Doxycycline in 5% dextrose was given once daily by oral gavage for the duration of the experiment to all groups. Reovirus was injected intratumorally as 1×10^5 plaque-forming units in a volume of 30 μL of PBS on day zero. Sham injection consisted of PBS only. GSK2606414 50 mg/kg or vehicle was administered via oral gavage once per day using a 5 days on, 2 days off schedule commencing on day zero. Vehicle was 0.5% hydroxypropyl methylcellulose and 0.1% Tween 80. Reovirus was injected intratumorally as 1×10^5 plaque-forming units in a volume of 30 μL of PBS on day zero and 1×10^7 in 30 μL of PBS on day nine. Sham injection consisted of PBS only. Tumor diameters were measured by vernier calipers, and volume was calculated as: (width × width × length)/2.

SUPPLEMENTAL INFORMATION

Supplemental Information can be found online at https://doi.org/10.1016/j.omto.2020.01.001.

AUTHOR CONTRIBUTIONS

Conceptualization, M.M. and K.J.H.; Methodology, M.M., M.P., V.R., K.F.B., H.G.S., J.K., and M.T.D.; Investigation, M.M., M.P., K.F.B., H.G.S., and H.W.; Writing and Review, M.M., H.P., R.V., A.M., and K.J.H.; Supervision, K.J.H.

CONFLICTS OF INTEREST

The authors declare no competing interests.

ACKNOWLEDGMENTS

This study was supported by The Oracle Cancer Trust (M.M.), The Oracle Cancer Trust Mark Donegan Fellowship (V.R. and J.K.), GetAhead (K.F.B.), the RM/ICR NIHR Biomedical Research Centre, Rosetrees Trust (grant numbers M48 and M444 to M.T.D. and K.J.H.), Cancer Research United Kingdom (M.P. and A.M.), and Anthony Long Charitable Trust (K.J.H.).

REFERENCES

1. Forrest, J.C., Campbell, J.A., Schelling, P., Stehle, T., and Dermody, T.S. (2003). Structure-function analysis of reovirus binding to junctional adhesion molecule 1. Implications for the mechanism of reovirus attachment. J. Biol. Chem. *278*, 48434–48444.
2. Maginnis, M.S., Forrest, J.C., Kopecky-Bromberg, S.A., Dickeson, S.K., Santoro, S.A., Zutter, M.M., Nemerow, G.R., Bergelson, J.M., and Dermody, T.S. (2006). Beta1 integrin mediates internalization of mammalian reovirus. J. Virol. *80*, 2760–2770.
3. Strong, J.E., Tang, D., and Lee, P.W. (1993). Evidence that the epidermal growth factor receptor on host cells confers reovirus infection efficiency. Virology *197*, 405–411.
4. Strong, J.E., Coffey, M.C., Tang, D., Sabinin, P., and Lee, P.W. (1998). The molecular basis of viral oncolysis: usurpation of the Ras signaling pathway by reovirus. EMBO J. *17*, 3351–3362.
5. Twigger, K., Roulstone, V., Kyula, J., Karapanagiotou, E.M., Syrigos, K.N., Morgan, R., White, C., Bhide, S., Nuovo, G., Coffey, M., et al. (2012). Reovirus exerts potent oncolytic effects in head and neck cancer cell lines that are independent of signalling in the EGFR pathway. BMC Cancer *12*, 368.
6. Smakman, N., van den Wollenberg, D.J.M., Borel Rinkes, I.H.M., Hoeben, R.C., and Kranenburg, O. (2005). Sensitization to apoptosis underlies KrasD12-dependent oncolysis of murine C26 colorectal carcinoma cells by reovirus T3D. J. Virol. *79*, 14981–14985.
7. Pakos-Zebrucka, K., Koryga, I., Mnich, K., Ljujic, M., Samali, A., and Gorman, A.M. (2016). The integrated stress response. EMBO Rep. *17*, 1374–1395.

8. Walter, P., and Ron, D. (2011). The unfolded protein response: from stress pathway to homeostatic regulation. Science *334*, 1081–1086.
9. Hetz, C., and Papa, F.R. (2018). The Unfolded Protein Response and Cell Fate Control. Mol. Cell *69*, 169–181.
10. Vattem, K.M., and Wek, R.C. (2004). Reinitiation involving upstream ORFs regulates ATF4 mRNA translation in mammalian cells. Proc. Natl. Acad. Sci. USA *101*, 11269–11274.
11. Chan, C.-P., Kok, K.-H., Tang, H.-M.V., Wong, C.-M., and Jin, D.-Y. (2013). Internal ribosome entry site-mediated translational regulation of ATF4 splice variant in mammalian unfolded protein response. Biochim. Biophys. Acta *1833*, 2165–2175.
12. Yoshida, H., Matsui, T., Yamamoto, A., Okada, T., and Mori, K. (2001). XBP1 mRNA is induced by ATF6 and spliced by IRE1 in response to ER stress to produce a highly active transcription factor. Cell *107*, 881–891.
13. Ye, J., Rawson, R.B., Komuro, R., Chen, X., Davé, U.P., Prywes, R., Brown, M.S., and Goldstein, J.L. (2000). ER stress induces cleavage of membrane-bound ATF6 by the same proteases that process SREBPs. Mol. Cell *6*, 1355–1364.
14. Smith, J.A., Schmechel, S.C., Williams, B.R.G., Silverman, R.H., and Schiff, L.A. (2005). Involvement of the interferon-regulated antiviral proteins PKR and RNase L in reovirus-induced shutoff of cellular translation. J. Virol. *79*, 2240–2250.
15. Kelly, K.R., Espitia, C.M., Mahalingam, D., Oyajobi, B.O., Coffey, M., Giles, F.J., Carew, J.S., and Nawrocki, S.T. (2012). Reovirus therapy stimulates endoplasmic reticular stress, NOXA induction, and augments bortezomib-mediated apoptosis in multiple myeloma. Oncogene *31*, 3023–3038.
16. Carew, J.S., Espitia, C.M., Zhao, W., Kelly, K.R., Coffey, M., Freeman, J.W., and Nawrocki, S.T. (2013). Reolysin is a novel reovirus-based agent that induces endoplasmic reticular stress-mediated apoptosis in pancreatic cancer. Cell Death Dis. *4*, e728.
17. Roulstone, V., Pedersen, M., Kyula, J., Mansfield, D., Khan, A.A., McEntee, G., Wilkinson, M., Karapanagiotou, E., Coffey, M., Marais, R., et al. (2015). BRAF- and MEK-Targeted Small Molecule Inhibitors Exert Enhanced Antimelanoma Effects in Combination With Oncolytic Reovirus Through ER Stress. Mol. Ther. *23*, 931–942.
18. Fernández de Castro, I., Zamora, P.F., Ooms, L., Fernández, J.J., Lai, C.M.H., Mainou, B.A., Dermody, T.S., and Risco, C. (2014). Reovirus forms neo-organelles for progeny particle assembly within reorganized cell membranes. mBio *5*, e00931-13.
19. Tenorio, R., Fernández de Castro, I., Knowlton, J.J., Zamora, P.F., Lee, C.H., Mainou, B.A., Dermody, T.S., and Risco, C. (2018). Reovirus σNS and μNS proteins remodel the endoplasmic reticulum to build replication neo-organelles. mBio. *9*, e01253-18.
20. Rogers, S.J., Box, C., Chambers, P., Barbachano, Y., Nutting, C.M., Rhŷs-Evans, P., Workman, P., Harrington, K.J., and Eccles, S.A. (2009). Determinants of response to epidermal growth factor receptor tyrosine kinase inhibition in squamous cell carcinoma of the head and neck. J. Pathol. *218*, 122–130.
21. Sakaue-Sawano, A., Kurokawa, H., Morimura, T., Hanyu, A., Hama, H., Osawa, H., Kashiwagi, S., Fukami, K., Miyata, T., Miyoshi, H., et al. (2008). Visualizing spatiotemporal dynamics of multicellular cell-cycle progression. Cell *132*, 487–498.
22. Wang, Y., Shen, J., Arenzana, N., Tirasophon, W., Kaufman, R.J., and Prywes, R. (2000). Activation of ATF6 and an ATF6 DNA binding site by the endoplasmic reticulum stress response. J. Biol. Chem. *275*, 27013–27020.
23. Smith, J.A., Schmechel, S.C., Raghavan, A., Abelson, M., Reilly, C., Katze, M.G., Kaufman, R.J., Bohjanen, P.R., and Schiff, L.A. (2006). Reovirus induces and benefits from an integrated cellular stress response. J. Virol. *80*, 2019–2033.
24. Dillon, M.T., Bergerhoff, K.F., Pedersen, M., Whittock, H., Crespo-Rodriguez, E., Patin, E.C., Pearson, A., Smith, H.G., Paget, J.T.E., Patel, R.R., et al. (2019). ATR Inhibition Potentiates the Radiation-induced Inflammatory Tumor Microenvironment. Clin. Cancer Res. *25*, 3392–3403.
25. Guthrie, L.N., Abiraman, K., Plyler, E.S., Sprenkle, N.T., Gibson, S.A., McFarland, B.C., Rajbhandari, R., Rowse, A.L., Benveniste, E.N., and Meares, G.P. (2016). Attenuation of PKR-like ER Kinase (PERK) Signaling Selectively Controls Endoplasmic Reticulum Stress-induced Inflammation Without Compromising Immunological Responses. J. Biol. Chem. *291*, 15830–15840.
26. He, H., Singh, I., Wek, S.A., Dey, S., Baird, T.D., Wek, R.C., and Georgiadis, M.M. (2014). Crystal structures of GCN2 protein kinase C-terminal domains suggest regulatory differences in yeast and mammals. J. Biol. Chem. *289*, 15023–15034.
27. Dey, M., Cao, C., Dar, A.C., Tamura, T., Ozato, K., Sicheri, F., and Dever, T.E. (2005). Mechanistic link between PKR dimerization, autophosphorylation, and eIF2alpha substrate recognition. Cell *122*, 901–913.
28. Cui, W., Li, J., Ron, D., and Sha, B. (2011). The structure of the PERK kinase domain suggests the mechanism for its activation. Acta Crystallogr. D Biol. Crystallogr. *67*, 423–428.
29. van Vliet, A.R., Giordano, F., Gerlo, S., Segura, I., Van Eygen, S., Molenberghs, G., Rocha, S., Houcine, A., Derua, R., Verfaillie, T., et al. (2017). The ER Stress Sensor PERK Coordinates ER-Plasma Membrane Contact Site Formation through Interaction with Filamin-A and F-Actin Remodeling. Mol. Cell *65*, 885–899.e6.
30. Lavoie, H., Thevakumaran, N., Gavory, G., Li, J.J., Padeganeh, A., Guiral, S., Duchaine, J., Mao, D.Y., Bouvier, M., Sicheri, F., and Therrien, M. (2013). Inhibitors that stabilize a closed RAF kinase domain conformation induce dimerization. Nat. Chem. Biol. *9*, 428–436.
31. Heidorn, S.J., Milagre, C., Whittaker, S., Nourry, A., Niculescu-Duvas, I., Dhomen, N., Hussain, J., Reis-Filho, J.S., Springer, C.J., Pritchard, C., and Marais, R. (2010). Kinase-dead BRAF and oncogenic RAS cooperate to drive tumor progression through CRAF. Cell *140*, 209–221.
32. Hatzivassiliou, G., Song, K., Yen, I., Brandhuber, B.J., Anderson, D.J., Alvarado, R., Ludlam, M.J., Stokoe, D., Gloor, S.L., Vigers, G., et al. (2010). RAF inhibitors prime wild-type RAF to activate the MAPK pathway and enhance growth. Nature *464*, 431–435.
33. Poulikakos, P.I., Zhang, C., Bollag, G., Shokat, K.M., and Rosen, N. (2010). RAF inhibitors transactivate RAF dimers and ERK signalling in cells with wild-type BRAF. Nature *464*, 427–430.
34. Rojas-Rivera, D., Delvaeye, T., Roelandt, R., Nerinckx, W., Augustyns, K., Vandenabeele, P., and Bertrand, M.J.M. (2017). When PERK inhibitors turn out to be new potent RIPK1 inhibitors: critical issues on the specificity and use of GSK2606414 and GSK2656157. Cell Death Differ. *24*, 1100–1110.
35. Mahameed, M., Wilhelm, T., Darawshi, O., Obiedat, A., Tommy, W.-S., Chintha, C., Schubert, T., Samali, A., Chevet, E., Eriksson, L.A., et al. (2019). The unfolded protein response modulators GSK2606414 and KIRA6 are potent KIT inhibitors. Cell Death Dis. *10*, 300.
36. Dillon, M.T., Barker, H.E., Pedersen, M., Hafsi, H., Bhide, S.A., Newbold, K.L., Nutting, C.M., McLaughlin, M., and Harrington, K.J. (2017). Radiosensitization by the ATR Inhibitor AZD6738 through Generation of Acentric Micronuclei. Mol. Cancer Ther. *16*, 25–34.
37. Twigger, K., Vidal, L., White, C.L., De Bono, J.S., Bhide, S., Coffey, M., Thompson, B., Vile, R.G., Heinemann, L., Pandha, H.S., et al. (2008). Enhanced in vitro and in vivo cytotoxicity of combined reovirus and radiotherapy. Clin. Cancer Res. *14*, 912–923.
38. Wiederschain, D., Wee, S., Chen, L., Loo, A., Yang, G., Huang, A., Chen, Y., Caponigro, G., Yao, Y.M., Lengauer, C., et al. (2009). Single-vector inducible lentiviral RNAi system for oncology target validation. Cell Cycle *8*, 498–504.

OMTO, Volume *16*

Supplemental Information

The PERK Inhibitor GSK2606414 Enhances Reovirus Infection in Head and Neck Squamous Cell Carcinoma via an ATF4-Dependent Mechanism

Martin McLaughlin, Malin Pedersen, Victoria Roulstone, Katharina F. Bergerhoff, Henry G. Smith, Harriet Whittock, Joan N. Kyula, Magnus T. Dillon, Hardev S. Pandha, Richard Vile, Alan A. Melcher, and Kevin J. Harrington

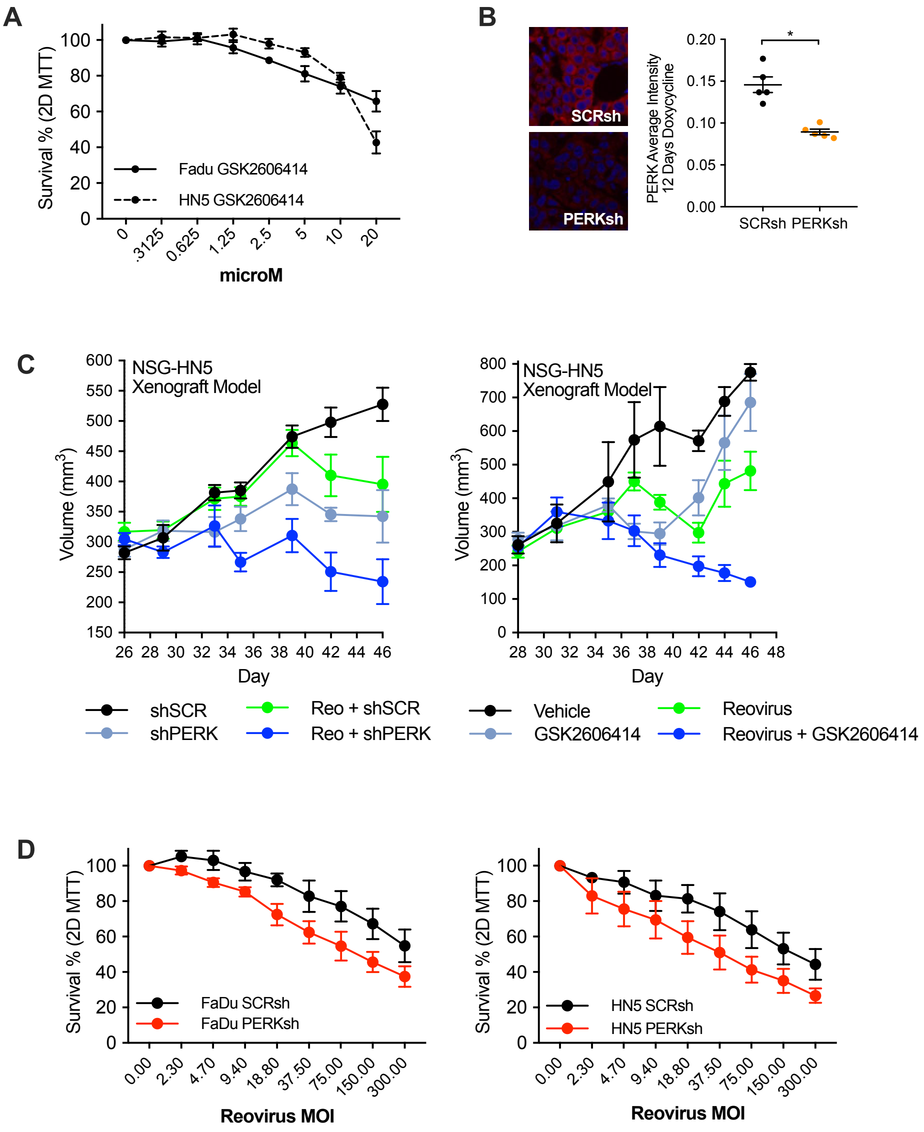

Supplementary Figure S1. Additional viability and growth curves to data in Figure 1. (A) Survival data for GSK2606414 alone in FaDu and HN5 cell lines measured at 72 h by MTT assay. (B) $2x10^6$ HN5 cells stably expressing lentiviral Tet-pLKO-puro were injected subcutaneously into NSG mice. HN5 cells contained scrambled shRNA (SCRsh) or PERK targeting shRNA (PERKsh). All mice received 50 mg/Kg doxycycline daily by gavage. After 12 days FFPE tumors were sectioned and stained by IHF to validate PERK knockdown. PERK levels were measured by automated image quantification using cell profiler. Example images of staining quality are shown for each. Data ±SEM with with statistical analysis by unpaired t-test *P < 0.05. Individual data points correspond to values for one tumor. (C) Corresponding volume data in mm^3 from figures 1F and 1G. (D) Survival as measured by MTT assay for reovirus in FaDu and HN5 cells with scrambled or PERK shRNA. Cells were treated with 400 g/mL doxycycline for 96 h before the addition of reovirus. Cells were maintained in doxycycline until the end of the assay at 72 h. Data shown in both A and D are from a minimum of three independent experiments.

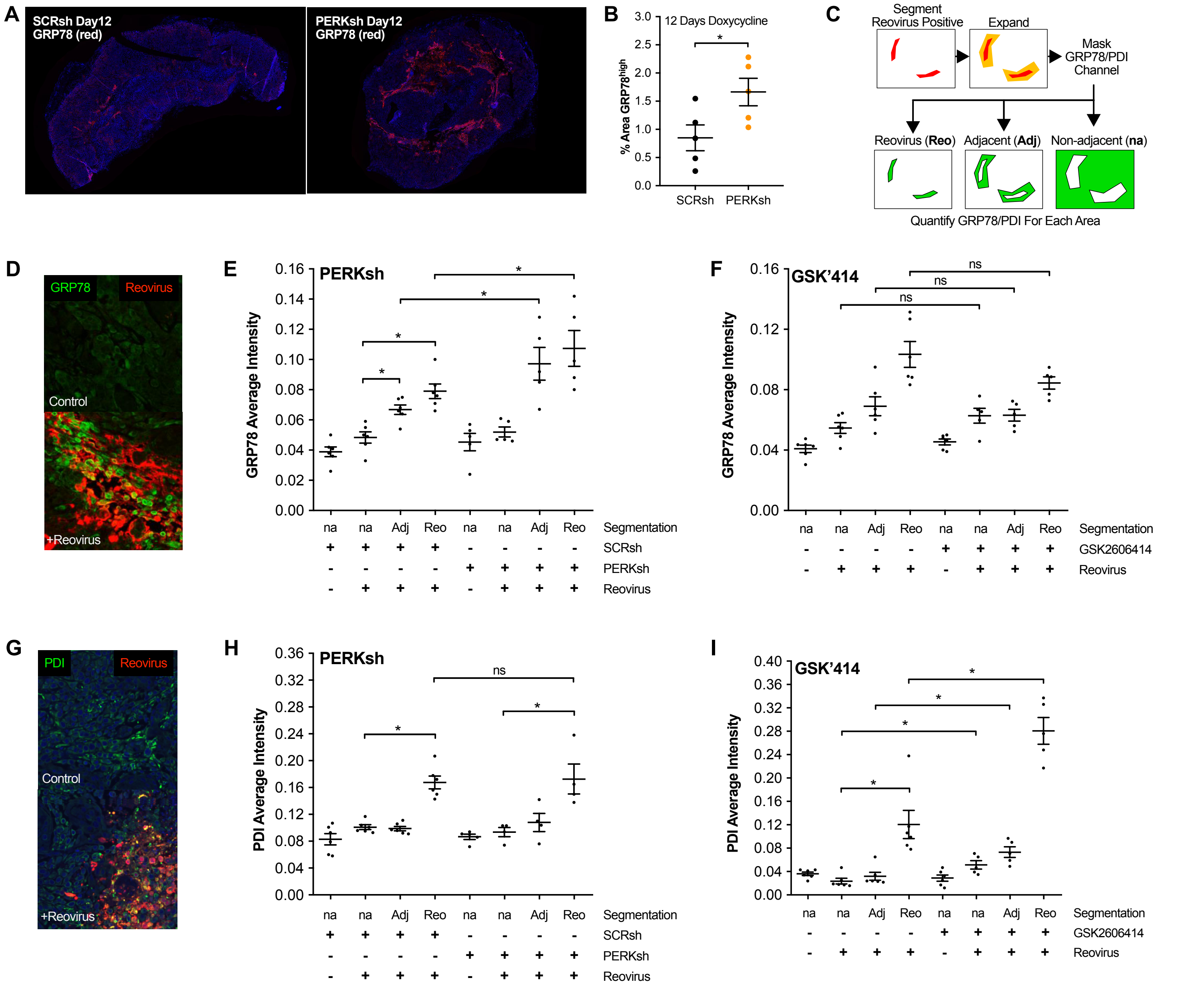

Supplementary Figure S2. Modulation of reovirus induced GRP78 and PDI *in vivo* by PERK knockdown or GSK2606414. (A) $2x10^6$ HN5 cells stably expressing lentiviral Tet-pLKO-puro were injected subcutaneously into NSG mice. HN5 cells contained scrambled shRNA (SCRsh) or PERK targeting shRNA (PERKsh). All mice received doxycycline daily by gavage. After 12 days FFPE tumors were sectioned and stained by IHF to assess changes to levels of the ER chaperone GRP78. (B) GRP78 high levels were quantified as in figure 3. (C) FFPE *in vivo* tumors at the end of experiments in figures 1F and 1G were co-stained for reovirus and GRP78 or PDI by IHF and imaged using the Vectra 3 imaging system from Perkin Elmer. Images were subject to automated image analysis using cell profiler. A schematic overview of the segmentation used in panels D-I to quantify GRP78 and PDI in areas positive for reovirus (Reo), adjacent to reovirus (Adj) and outside these two areas classified as non-adjacent (na). The area expanded by 10 microm around reovirus positivie areas equals approximately 1-2 cell widths immediately adjacent to reovirus infected areas with each Vectra image 525x700 microm in total. (D) Representative images of reovirus mu1C and GRP78 staining. (E,F) Comparison between the impact of PERK knockdown or GSK2606414 on GRP78 levels induced by reovirus infection. (G) Representative images of reovirus mu1C and PDI staining. (H,I) Comparison between the impact of PERK knockdown or GSK2606414 on PDI levels induced by reovirus infection. All panel data ±SEM with statistical analysis by unpaired t-test $*P < 0.05$. Individual data points correspond to values for one tumor.

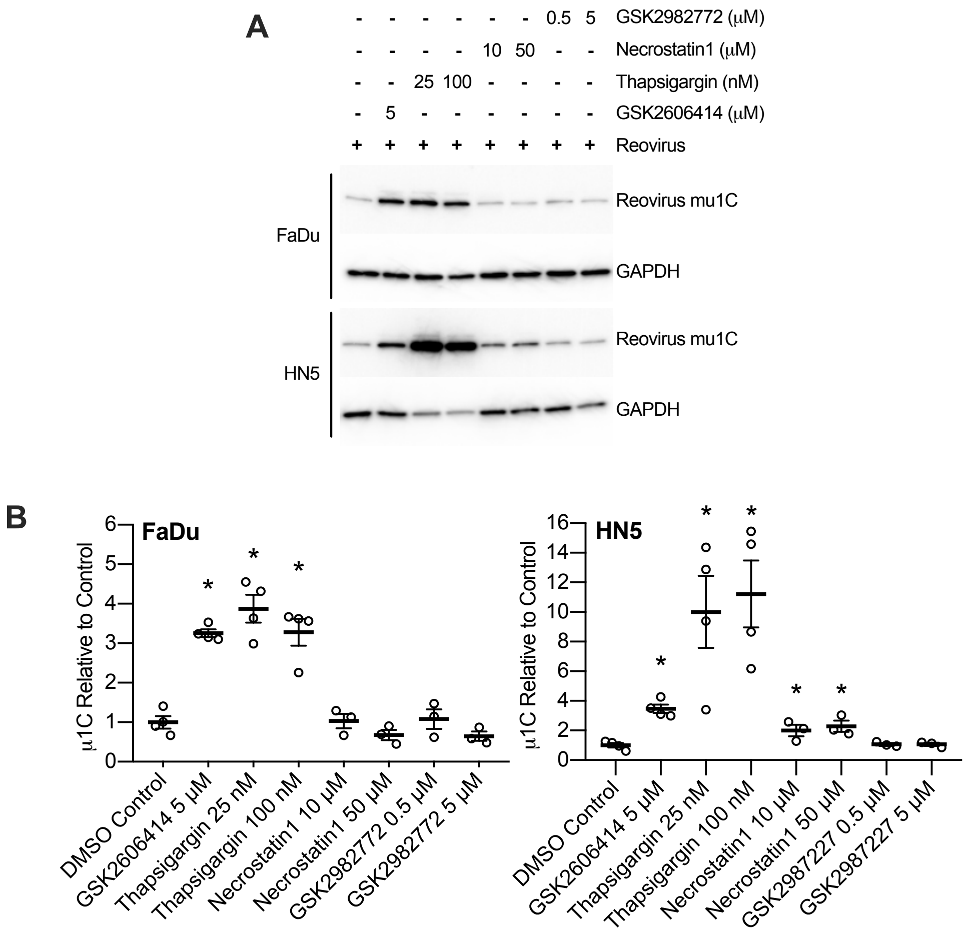

Supplementary Figure S3. Comparison of GSK2606414, Thapsigargin and RIPK1 inhibitors Necrostatin1 and GSK2982772 on reovirus capsid levels. (A) FaDu and HN5 cells were treated with reovirus in combination with DMSO only control, GSK2606414, or the RIPK1 inhibitors necrostatin1 and GSK2982772. Cell lysates were collected after 48 h and probed by western blot for the reovirus capsid protein mu1C. (B) Densitometry quantification of reovirus mu1C from western blots. Data is corrected for GAPDH levels and normalised to the average of controls. Data ±SEM of a minimum of three independent experiments with statistical analysis by unpaired t-test *$P < 0.05$.

Supplementary Table 1. Primers used for UPR live cell reporter cloning.

Reporter	Primer Name	Sequence
IRE1 endo	XBP1intron GFPFLAGforward	GTTGGATCCGCCACCATGCCAGTGGCCGGGTCTGCTGAGTCCGCAGCACTCAGAC TACGTGCACCTCTGCAGCAGGTGCAGGCCCAGTTGTCGTGAGCAAGGGCGAGGAG
IRE1 endo	XBP1intron GFPFLAGreverse	AATCAGGCGGCCGCTTACTTGTCGTCATCGTCTTTGTAGTCC
ATF4 prom	ATF4 forward	TTTCCAGAGTTTAGGAGGAGCCATG
ATF4 prom	ATF4 forward + 5' Overlap pHR	CAGAGATCCAGTTTGGATCGATAAGCTTGATATCGAATTCTTTCCAGAGTTTAGGAG GAGCCATG
ATF4 prom	ATF4 reverse	CATGTTGCGGTGCTTTGCTGGAA
ATF4 prom	ATF4 reverse + 3'Overlap mCherry	TGATGATGGCCATGTTATCCTCCTCGCCCTTGCTCACCATGTTGCGGTGCTTTGCTG GAA
ATF4 prom	mCherryMYC forward	ATGGTGAGCAAGGGCGAG
ATF4 prom	mCherryMYC reverse + 3' Overlap pHR	AGCTTGCATGCCTGCAGGTCGACTCTAGAGTCGCGGCCGCTTACAGATCCTCTTCT GAGATGAGTTTTTGTTCC
ATF6 bind	p5xATF6 forward	TAGGTCGAATTCCTCGAGACAGGTGCTGACG
ATF6 bind	p5xATF6 reverse	AATCAGGGATCCGGAGATCCTCTAGAGTCGACTCTAG

Supplementary Table 2. Primers for ATF4 transcript variants 1 and 2. Exon-Exon boundary indicated by /.

Variant	Primer Name	Sequence
2	ATF4 var2 forward	AGCCATGGC/GCTTCTCAC
	ATF4 var2 reverse	TGGAACACACAGCTACAGCA
1&2	ATF4 var1 and var2 forward	ATGGCGTATTAGGGGCAG
	ATF4 var1 and var2 reverse	AAGGCATCCT/CCTTGCTGTT
